# Manganese-deposited iron oxide promotes tumor-responsive ferroptosis that synergizes the apoptosis of cisplatin

**DOI:** 10.7150/thno.53346

**Published:** 2021-03-13

**Authors:** Junjie Cheng, Yang Zhu, Xin Xing, Jianmin Xiao, Hui Chen, Hongwei Zhang, Dan Wang, Yuanyuan Zhang, Guilong Zhang, Zhengyan Wu, Yangzhong Liu

**Affiliations:** 1 Department of Pharmacy, the First Affiliated Hospital of USTC, Division of Life Sciences and Medicine; Department of Chemistry, University of Science and Technology of China, Hefei 230001, China.; 2 Department of Stomatology, the First Affiliated Hospital of Wannan Medical College, Wuhu 241000, China.; 3 Key Laboratory of High Magnetic Field and Ion Beam Physical Biology, Hefei Institutes of Physical Science, Chinese Academy of Sciences, Hefei 230031, China.; 4 Department of Dental Implant Center, Stomatologic Hospital & College, Key Laboratory of Oral Diseases Research of Anhui Province, Anhui Medical University, Hefei 230032, China.; 5 School of Life Science, Anhui Medical University, Hefei 230032, China.; 6 School of Pharmacy, the Key Laboratory of Prescription Effect and Clinical Evaluation of State Administration of Traditional Chinese Medicine of China, Binzhou Medical University, Yantai 264003, China.

**Keywords:** cisplatin, ferroptosis, anticancer, theranostics, manganese

## Abstract

**Background:** Ferroptosis is a form of iron-dependent programmed cell death that differs from apoptosis with regards to both mechanism and cell morphology. Therefore, ferroptotic-based cancer therapy has shown significant potential to overcome the weaknesses of conventional therapeutics mediated by apoptosis pathways. Effective ferroptosis can be induced by the intracellular Fenton reaction that is dependent on the adequate supply of iron ions and H_2_O_2_ in cells. However, these are often insufficient due to intrinsic cellular regulation.

**Methods:** In this study, we designed a cisplatin prodrug-loaded manganese-deposited iron oxide nanoplatform (Pt-FMO) to trigger intracellular cascade reactions that lead to generation of reactive oxygen species (ROS) to enhance ferroptotic effect. The Pt-FMO causes the tumor microenvironment responsive to release manganese, iron ions and Pt-drugs. As manganese is an element that is able to catalyze the Fenton reaction more effectively than iron, coupled with the Pt-drugs that can promote generation of H_2_O_2_ in cells, the Pt-FMO is expected to significantly strengthen catalysis of the Fenton reaction, which favors the ferroptotic effect. Moreover, the Pt-drugs will eventually function as cisplatin. Thus, Pt-FMO is an ideal candidate for tumor ferroptotic combined with apoptotic treatment.

**Results:** *In vivo* results demonstrated that, at a dosage of only 8.89% Pt content, Pt-FMO is able to achieve a similar treatment effect as cisplatin. Hence, Pt-FMO exhibited significantly lower systemic toxicity compared to cisplatin. Additionally, Pt-FMO exhibits effective *T_2_*-weighted MRI enhancement for tumor imaging.

**Conclusion:** The Pt-FMO nanoplatform is designed to introduce mutual beneficial cascade reactions for promoting ferroptosis and apoptosis in combination with tumor MRI. The Pt-FMO system, which causes ferroptosis combined with apoptosis, can efficiently induce tumor cell death.

## Introduction

Ferroptosis is a non-apoptotic form of programmed cell death that has recently been discovered 1. It differs from apoptosis with regards to cell morphology, genetics and biochemistry, and is characterized by iron-dependent intracellular accumulation of lipid hydroperoxides (LPO) abundance 2. Although apoptosis-inducing drugs, such as cisplatin, are widely used in clinic for cancer chemotherapy, cancer cells are able to develop drug resistance by bypassing apoptosis. Therefore, non-apoptotic pathways are highlighted in the context of apoptotic resistance 3-5. Considering that ferroptosis has provided an alternative pathway to regular cell death, ferroptotic-based cancer therapy has shown great potential at eliminating the limitation of conventional therapeutics, mediated by apoptosis pathways 4.

It is well-known that ferroptosis is closely dependent on the level iron of in cells 1. Iron ions catalyze Fenton reaction of H_2_O_2_ and induce excessive ROS in cells, leading to accumulation of phospholipid peroxides, which eventually result in damage of intracellular proteins, lipids and DNA 4, 6-9. Usually, ferroptosis in cells is very low as the intrinsic cellular iron levels are strictly regulated by the iron-sensing system, while the endogenous H_2_O_2_ levels are limited. Supplying iron ions by iron-based nanomaterials in cells appears to be an effective approach for improving Fenton reaction. However, standalone iron-based nanomaterials are often insufficient as ferroptotic inducers as both high Fe doses (75 mg iron/kg for mice) and low pH levels (2-4) are required to efficiently generate ROS 10-12. Therefore, identifying more effective ferroptosis inducers is highly desired for future therapeutic applications 11, 13, 14.

Manganese (Mn) is another element that is able to effectively catalyze Fenton-like reaction and produce ROS at higher levels than iron 15. It is a biologically essential element that has good biocompatibility 16, 17. Due to its multivalence and high spin properties, manganese is one of the key cofactors of metalloenzymes that affect the metabolism and redox steady state of cells 18-20. Although very few studies have related ferroptosis with manganese ions alone, it has been found that Mn^2+^ ions that are released from manganese dioxide nanoparticles are able to convert endogenous H_2_O_2_ into toxic hydroxyl radicals (•OH) through Fenton-like reactions 15, 21, 22. This finding leads us to introduce Mn^2+^ into iron-based nanomaterials and constructed manganese carbonate-deposited iron oxide (FMO) nanoparticles to enhance ferroptosis. FMO is stable under physiological conditions, and it releases Mn^2+^ and Fe^2+^/Fe^3+^ ions under a weak acidic tumor environment. As ferroptosis is an iron-dependent process and associated with the Fenton reaction 1, 4, the combination of manganese and iron ions is able to enhance cell ferroptosis by providing iron for induction of ferroptosis and manganese for improvement of the Fenton reaction.

**Scheme 1:**
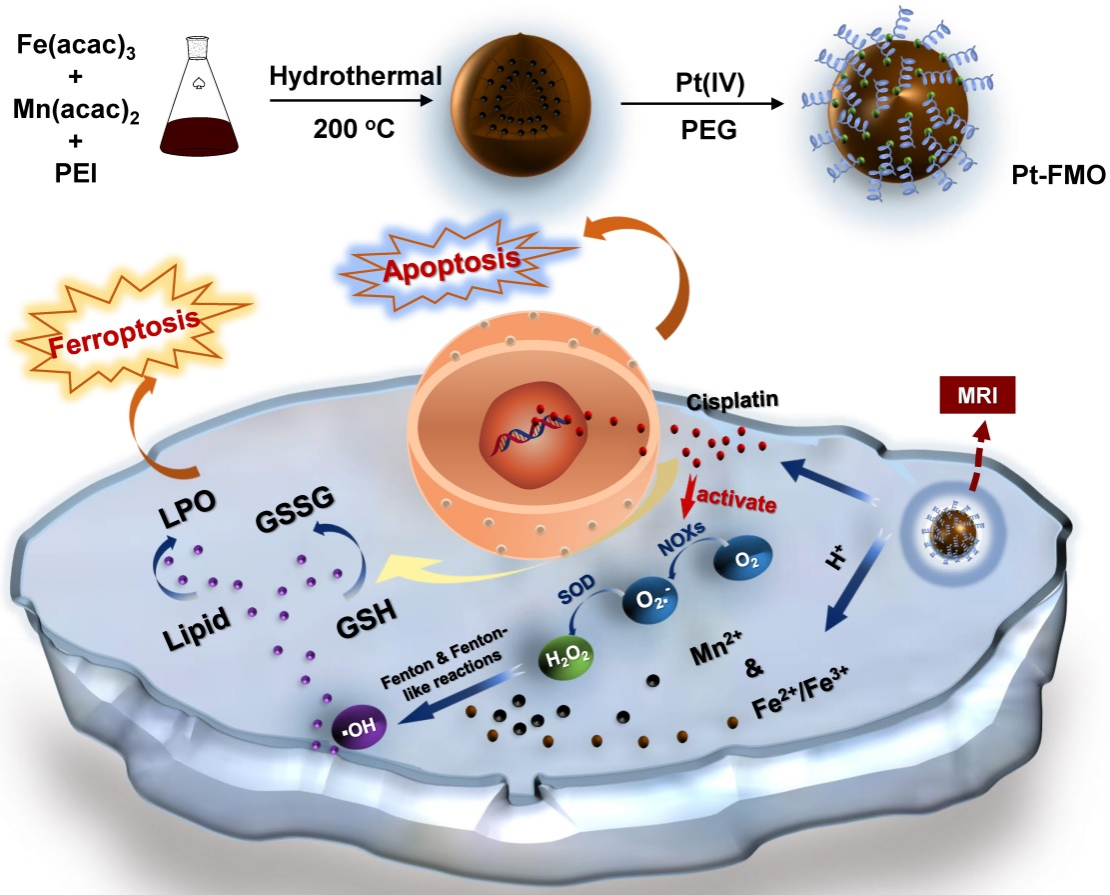
Schema of the preparation of magnetic Pt-FMO nanoparticles and its mechanism of inducing apoptosis and ferroptosis for the combined anti-tumor effect. The FMO vector sustainably releases Mn^2+^ and Fe^2+^/Fe^3+^ ions into the acidic environment, which promotes ferroptosis in cells via the Fenton and Fenton-like reactions. Endogenous GSH activates Pt(IV) prodrug and generates cytotoxic cisplatin, thus triggering cellular apoptosis. Meanwhile, cisplatin also mediates conversion of oxygen (O_2_) to generate downstream H_2_O_2_ that further elevates Fenton reactions.

Cancer cells tend to have a relatively favorable microenvironment for inducing Fenton or Fenton-like reactions as their abnormal metabolism causes weak acidity and high H_2_O_2_ levels 23. Nevertheless, H_2_O_2_ levels are balanced by intracellular reduction agents. Thus, elevated levels with persistent accumulation of intercellular H_2_O_2_ are still highly desired for the efficient Fenton reaction. In this study, a cisplatin prodrug (Pt(IV)) was conjugated to FMO, which forms a cascade reaction nanoplatform, Pt-FMO (Scheme 1). The low cytotoxic Pt(IV) prodrug is able to activated by intracellular reducing agents such as glutathione (GSH), that yield high cytotoxic cisplatin within tumor cells. This drug activation process is able decrease cellular GSH levels, which may help benefit the Fenton reaction as GSH is a well-known ROS scavenger. More importantly, cisplatin is capable of triggering the generation of H_2_O_2_ in cells during its action, which causes upregulation of intracellular H_2_O_2_ 24, 25. Therefore, this Pt-FMO nanoplatform triggers cascade reactions through the use of a platinum drug in order to up-regulate intracellular H_2_O_2,_ which can be converted to highly toxic •OH through catalysis of Fenton reaction by manganese/iron ions that are released from FMO, and further efficiently promote ferroptosis in cancer cells.

In addition to promoting cellular ferroptosis using Pt-FMO, cisplatin in Pt-FMO is an effective anticancer drug that is widely used in clinic and triggers cell apoptosis by damaged DNA. Approximately 40-80% of all multitudinous cancer patients have been treated by cisplatin or its related analogues 26-28. However, application of platinum-based anticancer drugs has been limited due to their severe side-effects and drug resistance 29, 30. The mechanism of resistance is closely related to self-protection of cancer cells, and includes reduced intracellular drug accumulation, increased drug detoxification, and altered DNA damage induced apoptosis pathways, in order to combat chemotherapy-induced apoptosis 29, 31-34. Therefore, this Pt-FMO nanoplatform not only provides a combination therapy of ferroptosis and apoptosis, but also supplies an extra non-apoptotic pathway that eliminates the limitation of traditional cisplatin therapeutics that are mediated by apoptotic pathways. Moreover, FMO has effective MRI contrast ability, which allows Pt-FMO to be a convenient nanoplatform for theranostic application.

## Results and Discussion

### Synthesis and characterization of Pt-FMO

**Figure 1:**
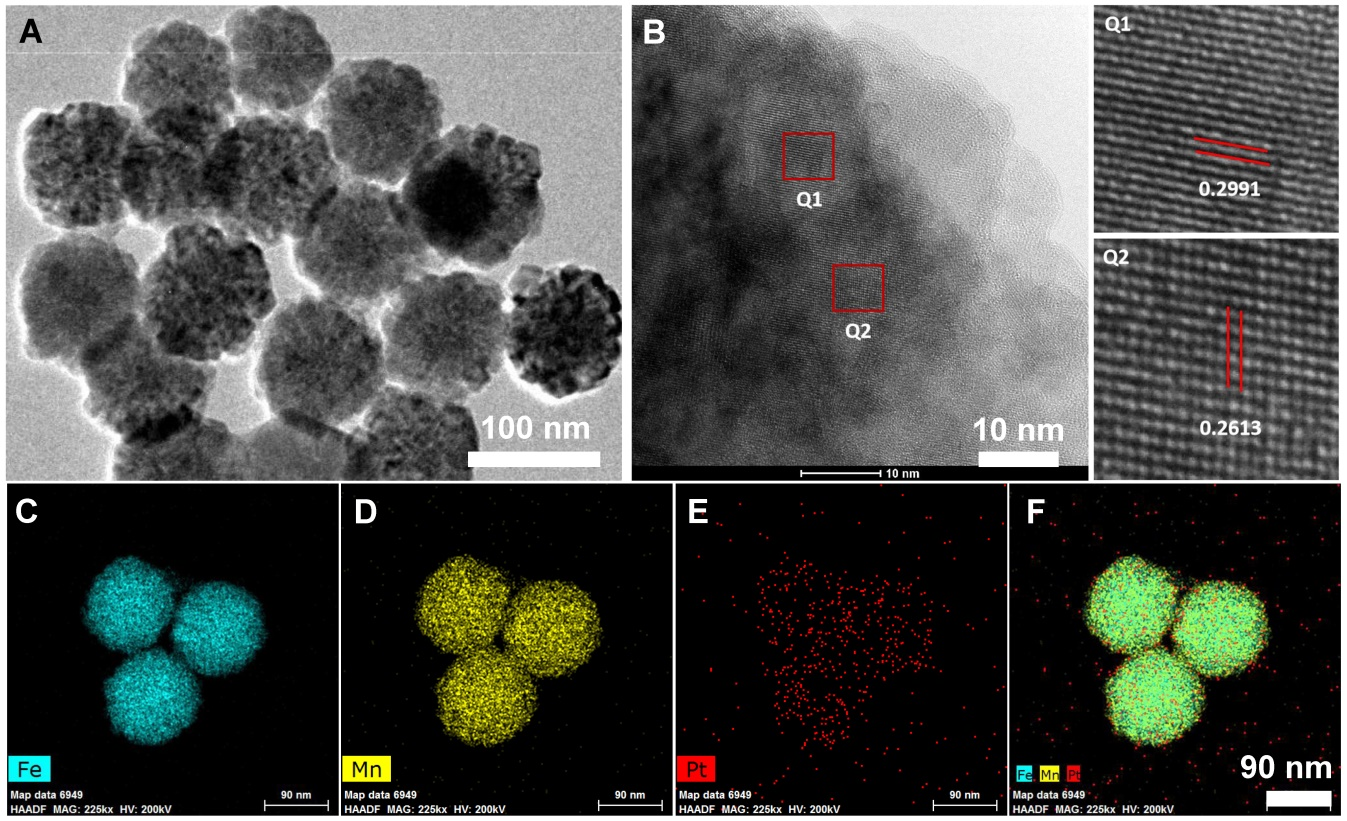
Characterization of Pt-FMO nanoparticles using transmission electron microscopy (TEM). (A) TEM image of Pt-FMO. (B) High resolution TEM images of Pt-FMO. Q1 corresponds to the (311) plane of Fe_3_O_4_ and Q2 corresponds to the (006) plane of MnCO_3_. (C-F) Energy dispersive spectrometer (EDS) mapping images of Fe (C), Mn (D), Pt (E), and merged imaging (F).

FMO nanoparticles were synthesized according to previously published literature *via* solvothermal reactions using iron acetylacetonate and manganese acetylacetonate as precursors 35. Subsequently, FMO was modified by polyethylenimine (PEI) to increase colloidal stability. Then, a cisplatin prodrug (Pt(IV)), *c,c,t*-[Pt(NH_3_)_2_Cl_2_(O_2_CCH_2_CH_2_CO_2_H)_2_] (Figure S1), was conjugated to the surface of FMO through amide bonds, which led to the formation of Pt-FMO (Scheme 1). Transmission electron microscopy (TEM) image demonstrated that Pt-FMO had a spherical shape with a uniform size of approximately 90 nm (Figure 1A, S2). Additionally, FMO presented a nanocluster structure that was comprised of tiny crystals (Figure 1B). The enlarged square areas (Q1 and Q2) in Figure 1B clearly depict the lattice fringes of Pt-FMO. Two types of interplanar spacing, 0.2991 nm (in Q1) and 0.2613 nm (in Q2) correspond to the (311) plane of Fe_3_O_4_ (PDF: 39-1346) 36 and the (006) plane of MnCO_3_ (PDF:44-1472) 37, 38. These results clearly demonstrate that the FMO is composed of Fe_3_O_4_ and MnCO_3_. The energy dispersive X-ray element mapping (Figure 1C-F) and spectroscopic analysis (Figure S3) confirmed the presence of Fe, Mn and Pt elements within the Pt-FMO nanoparticle. Energy dispersive spectrometer (EDS) mapping exhibited that the Fe, Mn and Pt elements were homogeneously distributed into Pt-FMO (Figure 1C-F). This result confirmed the uniform deposition of manganese into FMO nanoparticles, and also indicated the successful conjugation of Pt(IV) prodrugs to FMO.

The dynamic light scattering (DLS) measurement indicated that the hydrodynamic size of Pt-FMO was approximately 200 nm, due presence of both PEI and polyethylene glycol (PEG) on the surface of these particles (Figure S4A). The zeta potential of Pt-FMO was significantly decreased compared to FMO, due to conjugation of the negatively charged Pt(IV) prodrug to FMO (Figure S4B), which further confirms the successful loading of Pt(IV) prodrugs to FMO. Moreover, the stability measurement indicated that the Pt-FMO was stable during the 48 h incubation within the cell culture medium that contained 10% fetal bovine serum (Figure S5).

**Figure 2:**
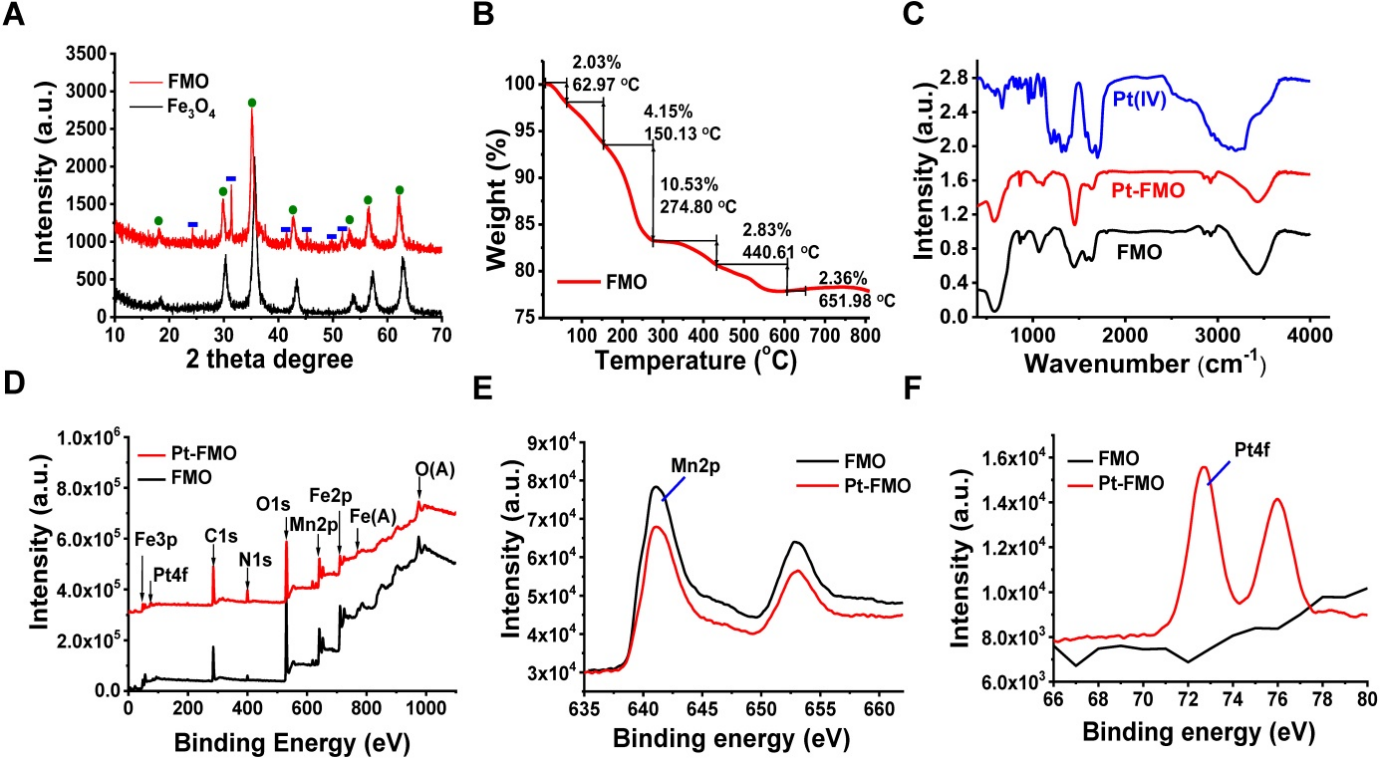
Physicochemical characterization of nanoparticles. (A) XRD patterns of FMO and Fe_3_O_4_. The green dots represent diffraction peaks of Fe_3_O_4_, while the blue squares represent MnCO_3_ peaks. (B) TGA analysis of FMO. (C) FT-IR spectra of Pt(IV), FMO and Pt-FMO. (D) XPS spectra of FMO and Pt-FMO. (E-F) The expanded regions of XPS spectra showing the peaks of Mn 2p (E) and Pt 4f (F) of FMO and Pt-FMO.

The X-ray crystal diffraction (XRD) spectrum of FMO (Figure 2A) demonstrates the typical diffraction peaks of nanocomposites that contain Fe_3_O_4_ (green dots) and MnCO_3_ (blue rectangles). In addition, the diffraction peaks of MnCO_3_ in FMO were assigned to the calcite structure of rhodochrosite (PDF: 44-1472). On the other hand, Fe_3_O_4_ was the spinel structure of magnetite (PDF: 39-1346). These results validate the successful deposition of MnCO_3_ into Fe_3_O_4_ nanoparticles 39. Furthermore, the diffraction peak of FMO has slightly shifted to the left, which is likely attributed to the intercalation effect of MnCO_3_ that increases interplanar spacing of FMO. The PEI content in FMO has a key function in conjugated Pt(IV) prodrugs and can be calculated using a thermogravimetric analyzer (TGA). The weight loss prior to 150 °C was generally due to a loss of water molecules, including adsorbed water and bound water. Approximately 15.72% of the weight was lost between 150 °C and 650 °C, likely due to the burning of the PEI chain (Figure 2B). This result demonstrated that the existence of large amount of PEI in FMO was a large amount of PEI in FMO, which allows effective conjugation of FMO with Pt (IV) prodrugs.

Subsequently, conjugation of Pt(IV) to FMO can be detected through the Fourier transform infrared spectroscopy (FT-IR) (Figure 2C). The curve of Pt(IV) shows strong peaks at 1720 cm^-1^, as well as a broad peak at 3000-3500 cm^-1^, which originates from both the bending vibration and stretching vibration of -COOH in Pt(IV). Remarkably, the strong characteristic peak of -COOH at 1720 cm^-1^ of Pt(IV) prodrug disappears in Pt-FMO, which indicates covalent conjugation of Pt(IV) to FMO nanoparticles through an amide bond. Furthermore, successful loading of Pt(IV) prodrugs on FMO can be verified using X-ray photoelectron spectroscopy (XPS) analysis. The full XPS spectra of FMO confirms the presence of Fe, Mn, C, N and O elements within the FMO nanoparticles (Figure 2D). In addition, the binding energy of Mn 2p demonstrated a dominant peak at 641 eV, which is consistent with the divalent state of Mn ions of MnCO_3_ in FMO 40, 41 (Figure 2E). Compared to FMO, the XPS spectra of Pt-FMO shows an obviously new signal at 72.5 eV, which is assigned to the binding energy of tetravalent Pt (Figure 2D and F) 42, suggesting that Pt(IV) prodrug is successfully conjugated to FMO.

Following that, the release behavior of Fe, Mn and Pt ions was assessed (Figure S6). It is worth noting that, despite the release of all components (Fe, Mn and Pt) was promoted by the acidic environment (pH 4.6), Mn^2+^ demonstrated the strongest dependency of pH, likely due to the fact that MnCO_3_ is more sensitive to the acidic environment. Furthermore, Pt also demonstrated a pH-dependent release, which can be caused by disassociation of FMO, which sheds the payload of Pt(IV) prodrug. In order to confirm the catalytic activity of Pt-FMO within the tumor microenvironment, the generation of •OH *via* Fenton or Fenton-like reaction was measured using electron spin resonance (ESR) spectroscopy through the use of a spin trapper 5,5-Dimethyl-1-pyrroline N-oxide (DMPO). The ESR spectrum clearly demonstrated a characteristic quartet signal (1:2:2:1) of DMPO-OH at both pH 6.5 and 4.6, indicating the generation of •OH species *via* Pt-FMO-mediated catalysis under acidic conditions (Figure S7). The observation suggests that Pt-FMO is able to perform catalytic activity of the Fenton reaction in the acidic tumor microenvironment, and therefore, may be a candidate ferroptotic agent for stimuli-responsive tumor treatment.

### Cellular uptake

**Figure 3:**
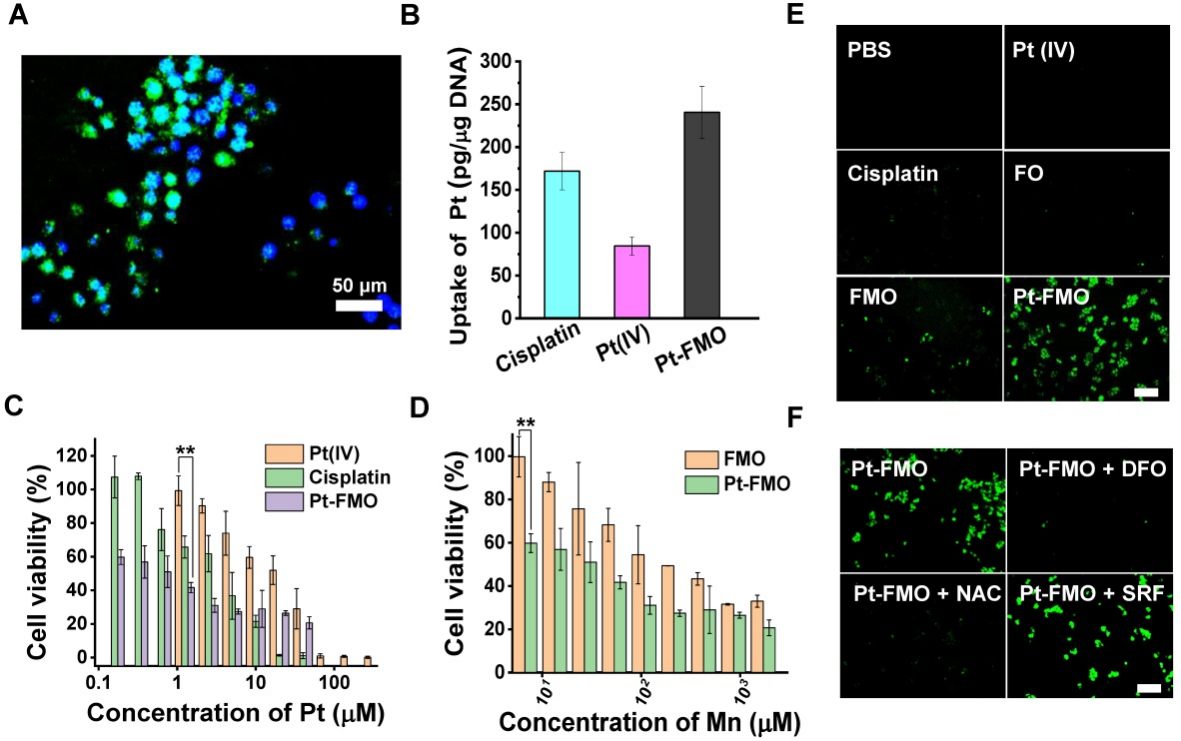
Cellular uptake, toxicity and ROS generation of Pt-FMO. (A) Fluorescence microscopy images of HeLa cells post-incubation with FMO-FITC for 4 h at 37 °C. Images depict merged images of FITC (green) and nuclear Hoechst staining (blue). (B) DNA platination of HeLa cells after incubation with cisplatin, Pt(IV) or Pt-FMO at an equivalent concentration of 40 µM Pt for 6 h at 37 °C. (C-D) Cell viability of HeLa cells post-treatment with cisplatin, Pt(IV), FMO and Pt-FMO for 48 h at 37 °C; n = 6; *p < 0.05, **p < 0.01, ***p < 0.001. Statistical analyses were conducted using t-test. (E) Analysis of ROS levels in HeLa cells using DCFH-DA staining. Cells were incubated with cisplatin, Pt(IV) or Pt-FMO at the equivalent Pt concentration of 15 µM for 4 h at 37 °C. Scale bar represents 200 µm. (F) Modulation of ROS levels in cells by the ferroptosis inhibitor N-acetyl-L-cysteine (NAC), Deferoxamine (DFO) and the ferroptosis promoter Sorafenib (SRF). DFO (100 µM) and NAC (3 mM) were separately used to pretreat cells for 1 h. SRF (3 µM) and Pt-FMO was added at the same time. Scale bar represents 200 µm.

It is well-known that cisplatin acts through four key steps: cellular uptake, equation/activation, DNA binding, and DNA lesions that lead to cell death 43. In order to investigate the cellular uptake of nanoparticles, *in vitro* cellular internalization was conducted utilizing fluorescein isothiocyanate (FITC)-labeled FMO (FMO-FITC) in cells *via* fluorescence microscopy. As depicted in Figure 3A, bright-green signals were present in HeLa cells, which verified that FMO-FITC is internalized by cells (Figure S8). Additionally, the DNA platination of Pt-FMO was subjected to inductively couple plasma-mass spectrometry (ICP-MS) to gain further insight into the molecular targeting activity of Pt-FMO. Results indicated that DNA platination by Pt-FMO in HeLa cells (approximately 240.49 pg/μg DNA) was significantly higher compared to Pt(IV) (84.44 pg/μg DNA) or cisplatin (171.90 pg/μg DNA) (Figure 3B), which indicates that the covalent conjugation of Pt(IV) to FMO nanoparticle favors internalization of platinum-drugs by cells. This further enhances the binding efficiency of Pt-FMO to genomic DNA in cancer cells and is expected to improve the anti-tumor activity of Pt-FMO.

### Mechanism underlying Pt-FMO-induced cell Ferroptosis

Non-ferrous elements, which include manganese (Mn), copper (Cu) and cobalt (Co), are able to catalyze Fenton-like reactions to generate ROS, and can therefore be considered as alternatives to iron for induction of ferroptosis 14, 44. In order to further investigate the anti-cancer mechanism of Pt-FMO, the *in vitro* cytotoxicity effect was evaluated in HeLa cervical cancer cells as cisplatin is the first-line chemotherapeutic drug in cervical cancer 45. As indicated in Figure S9, the single component Fe_3_O_4_ nanoparticle (FO) that is synthesized within this study has low cytotoxicity. However, cell viability was found to be clearly reduced post-treatment with FMO. This result is consistent with the hypothesis that Mn is able to catalyze Fenton-like reaction in cells more efficiently than iron and produce ROS that allow killing of tumor cells. Moreover, cell proliferation was further decreased by Pt-FMO treatment, which implies favorable combination of FMO and Pt(IV) prodrug therapies (Figure 3C-D). Interestingly, Pt-FMO apparently demonstrates greater anticancer efficacy relative to free cisplatin at low concentrations. In order to further validate this finding, the live/dead stain assay was conducted to better visualize death of tumor cells (Figure S10). It was obvious that the majority of cells were dead post-treatment with cisplatin, and complete cell death was observed in the Pt-FMO treated group. This remarkable anti-tumor performance of Pt-FMO could be attributed to an efficient Pt delivery capability of Pt-FMO and adequate ROS induced by Mn^2+^ catalytic Fenton-like reactions.

**Figure 4:**
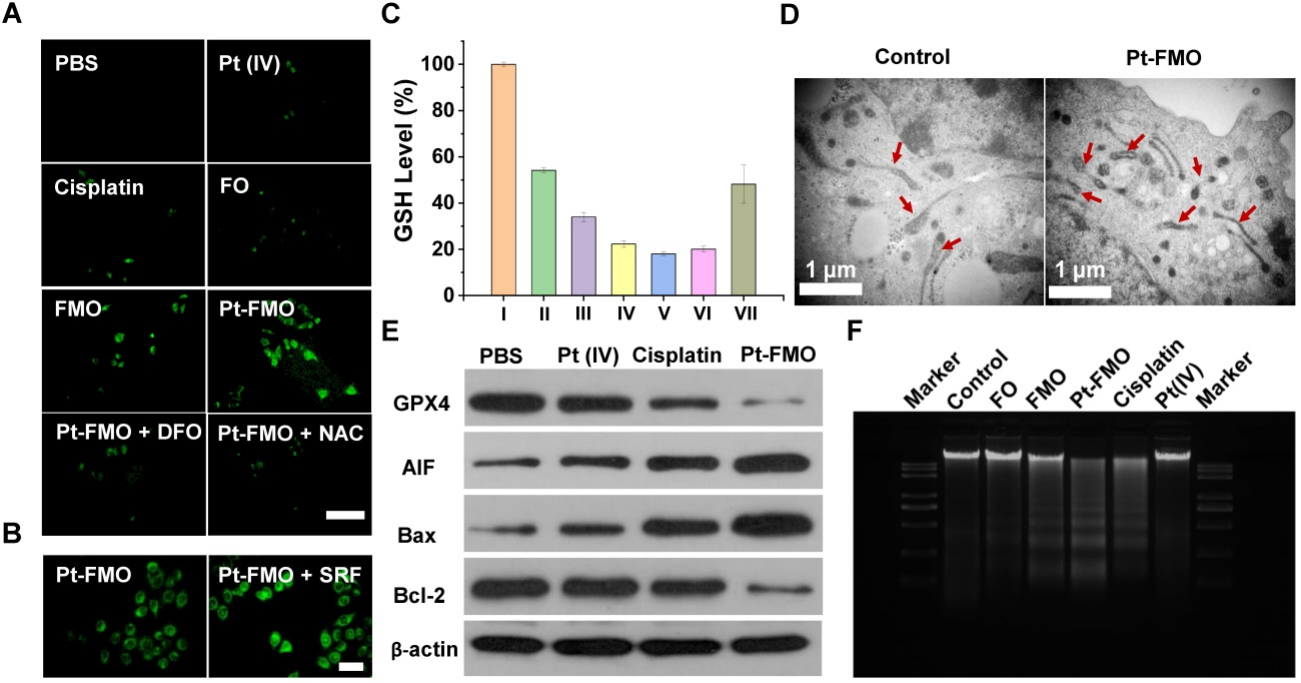
Cellular assays of Pt-FMO-mediated ferroptosis and apoptosis. (A) Lipid peroxide measured by BODIPY-C11 staining in HeLa cells. Cells were incubated with either cisplatin, Pt(IV) or Pt-FMO at an equivalent Pt concentration of 15 µM for 4h at 37 °C. Next, DFO (100 µM) and NAC (3 mM) were separately used to pretreat cells for 1 h. Scale bar represents 100 µm. (B) Modulation of lipid peroxide levels by the ferroptosis promoter SRF. Cells were incubated with Pt-FMO at an equivalent Pt concentration of 15 µM for 4h at 37 °C. SRF (3 µM) and Pt-FMO was added at the same time. Scale bar represents 100 µm. (C) Intracellular GSH levels of HeLa cells post-treatment with I: control; II: Pt-FMO (2.5 µM); III: Pt-FMO (5 µM); IV: Pt-FMO (10 µM); V: Pt-FMO (20 µM); VI: Pt-FMO (10 µM) plus SRF; VII: Pt-FMO (10 µM) plus DFO. SRF and Pt-FMO was added at the same time. DFO was used separately to pretreat cells for 1 h. (D) TEM imaging of cellular morphology with treatment of Pt-FMO (10 µM Pt). (E) Western blot analysis of GPX4, AIF, Bax and Bcl-2 expression in HeLa cells post-treatment with PBS, Pt(IV), cisplatin and Pt-FMO at an equivalent Pt concentration at 20 µM. (F) DNA laddering assay for evaluation of DNA damage in HeLa cells after incubation with FO, FMO, Pt-FMO, cisplatin and Pt(IV). Cells were incubated with cisplatin, Pt(IV) or Pt-FMO at an equivalent Pt concentration of 20 µM for 24h at 37 °C. The concentration of Fe and Mn in FO and FMO were consistent with that in Pt-FMO.

Next, we investigated the cytotoxic mechanisms of Pt-FMO, including cellular response such as ROS generation, lipid peroxidation, GSH depletion, mitochondria shrinkage, protein expression and DNA damage. Encouraged by the results of catalytic activities of FMO in solution, we first assessed the cellular ROS levels using a 2',7'-dichlorofluorescin diacetate (DCFH-DA) staining assay, which exhibited green fluorescence under the presence of ROS. We also found slight green fluorescence after FO treatment, thus confirming the low efficacy of Fenton reaction catalyzed by Fe_3_O_4_. In comparison, FMO treatment resulted in clear green fluorescence, and cells incubated with Pt-FMO showed significantly bright fluorescence, which reveals increasing accumulation of •OH (Figure 3E, S11). This data provides clear evidence that FMO leads to efficient ROS generation through Fenton-like reactions, and that this effect can be further enhanced by participation of Pt(IV) prodrugs. As the execution of ferroptosis requires accumulation of cellular ROS in an iron-dependent manner, 46, 47 a ROS modulator and ferroptosis regulator were introduced to determine the mechanism underlying Pt-FMO-induced cell death. As shown in Figure 4B, ROS production was alleviated in the presence of either the ROS scavenger (N-acetyl-L-cysteine, NAC) or ferroptosis inhibitor (Deferoxamine, DFO), while treatment with ferroptosis activator (Sorafenib, SRF) further enhanced ROS levels (Figure 3F, S12), which suggests that ROS generation is associated with the ferroptosis pathway.

It is well-known that three crucial events occur during the process of ferroptosis, including lipid peroxidation, GSH depletion and GPX4 downregulation 4. In order to investigate the ferroptosis-inducing activity of Pt-FMO in tumor cells, we characterized the levels of intracellular lipid peroxide (LPO), which is a key hallmark of ferroptosis 7. The lipid peroxidation probe BODIPY-C11 was utilized to quantify the lipid peroxide levels. Cells treated with Pt-FMO displayed the brightest green fluorescence, thus validating that Pt-FMO is associated with a significant increase in LPO level (Figure 4A). The LPO levels were also examined in the presence of NAC, DFO and SRF. Similar to the ROS results, NAC and DFO led to a remarkable reduction in cellular LPO levels, whereas SRF significantly improved LPO levels (Figure 4A-D, S13-14). These results provide clear evidence of Pt-FMO-induced ferroptosis.

Next, we quantified the capability of Pt-FMO to deplete GSH *via* the 5,5'-Dithiobis(2-nitrobenzoic acid) (DTNB) assay. Our results indicated that the treatment of Pt-FMO significantly lowered GSH levels in cells in a concentration-dependent manner (Figure 4C). Additionally, Pt-FMO containing 20 μM Pt led to a reduction of GSH levels down to 18.0% relative to the untreated group. The treatment of SRF further enhanced GSH depletion, whereas addition of DFO significantly mitigated GSH depletion caused by Pt-FMO. It is well-known that GPX4 has a crucial role in the cellular lipid repair system, and can be inactivated by depletion of GSH 10. Based on the effective LPO induction and high GSH depletion capability of the Pt-FMO, intracellular GPX4 levels were further monitored using Western blot. Both cisplatin and Pt-FMO can decrease GPX4 levels, while Pt-FMO demonstrated significantly higher inhibition efficiency compared to cisplatin (Figure 4D). These results are consistent with the literature that cisplatin can induce weak ferroptosis 48. In addition, cisplatin is capable of up-regulating H_2_O_2_ levels in tumor cells (Figure S15), which subsequently favors ROS generation catalyzed by FMO. Therefore, a greater inhibition of GPX4 was identified in the Pt-FMO treatment group. Moreover, overexpression of the apoptosis-inducing factor (AIF) was seen in the Pt-FMO treated group (Figure 4E). As AIF can be up-regulated by lipid peroxidation 47, 49, this result further confirms that lipid peroxidation induces ferroptosis. Additionally, as one of the characteristic consequences of ferroptosis, TEM imaging clearly shows mitochondrial shrinkage in the cells treated with Pt-FMO (Figure 4E), which can be found in SRF (a well-known ferroptotic agent) treated group (Figure S16). Inspired by the above experimental results, the ferroptosis inhibitor and activators were introduced into the Pt-FMO treatment in order to investigate their effects on cytotoxicity (Figure S17). As expected, pretreatment using an ferroptosis inhibitor, NAC or DFO, significantly restored the cell variability suppressed by Pt-FMO. On the other hand, the treatment of ferroptosis enhancer SRF improved the inhibitory activity of Pt-FMO. Statistical analysis demonstrated the significant differences of these cell viability values. This data clearly indicates that ferroptosis is involved in the Pt-FMO-induced cell death pathway.

For further mechanistic investigation, the expression of Bcl-2 family proteins, including the pro-apoptotic protein (Bax) and the antiapoptotic protein (Bcl-2) were analyzed to support the combined ferroptotic/apoptotic effects of Pt-FMO. It can be shown that expression of Bax led to an increase in a trend with treatment of Pt(IV), cisplatin and Pt-FMO, while expression of Bcl-2 was reversed. These results clearly demonstrate that Pt-FMO can not only induce ferroptosis, but also apoptosis. Moreover, the DNA laddering assay was carried out to assess DNA damage. Results demonstrated that cisplatin-treated HeLa cells produced a visually discernible DNA ladder, which could be attributed to the highly apoptotic effect of cisplatin (Figure 4F). Comparatively, we could only find a slight DNA ladder in cells treated with Pt(IV) and FO, which suggests the negligible pro-apoptotic activity of FO and low-dose Pt(IV). FMO can also induce the DNA ladder, which might be due to its LPO activation that causes DNA fragmentation 49, 50. A significantly larger amount of DNA fragments were observed in cells treated with Pt-FMO than that with cisplatin or FMO alone, which shows the remarkable combinatory effect of Pt-FMO.

### *In vivo* antitumor assay

**Figure 5:**
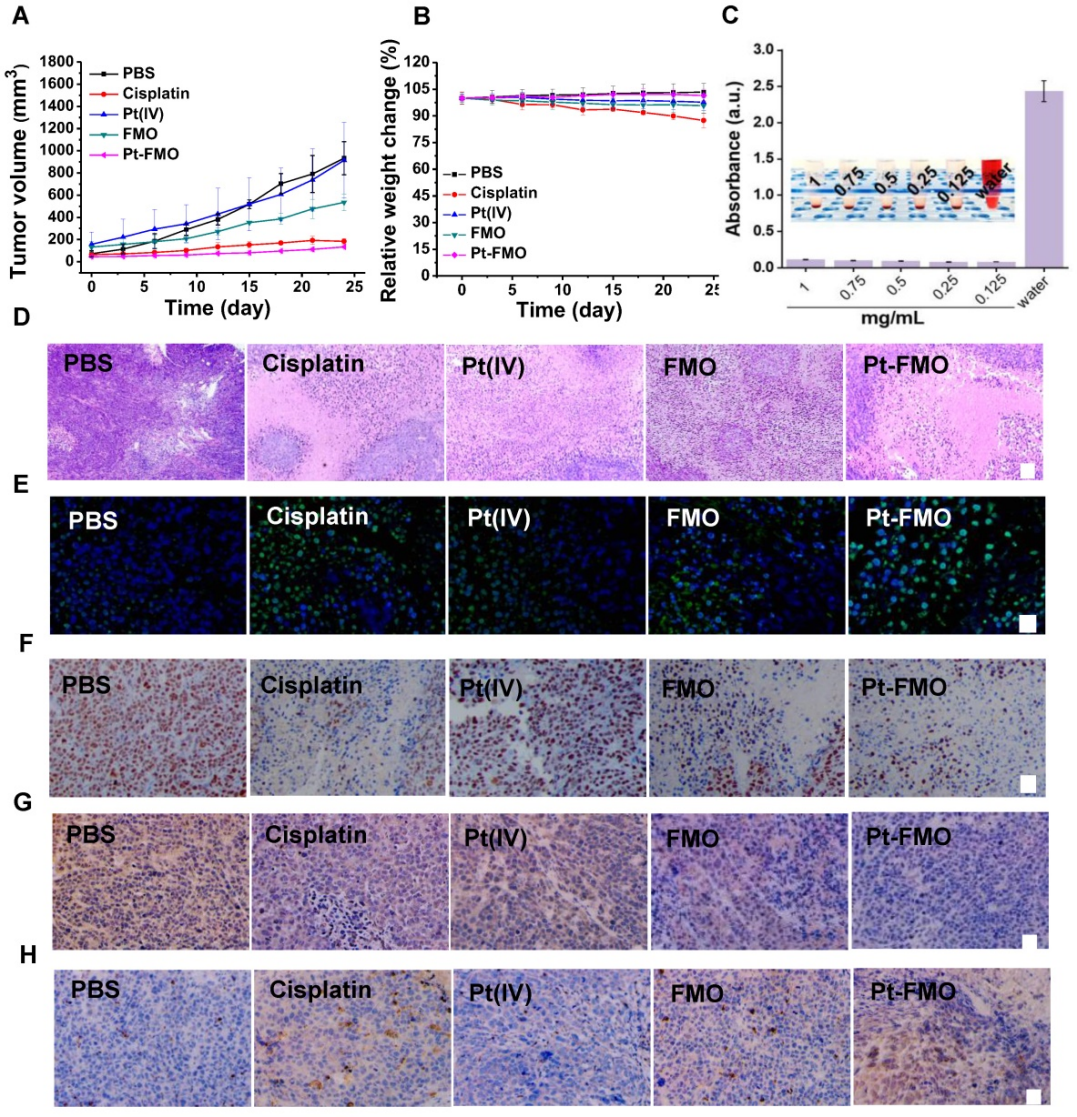
*In vivo* antitumor effect of Pt-FMO. The tumor-bearing BALB/c-Nude mice were treated with either PBS, free cisplatin, free Pt(IV), FMO or Pt-FMO at a dose of 5 mg/kg *via* tail vein injection. (A) Tumor volume growth curves. The BALB/c-Nude mice bearing HeLa tumors were treated with various agents. (B) Body weight of nude mice. (C) Hemolysis assay of Pt-FMO at various concentrations. The UV-vis spectra absorbance of the supernatant of red blood cells at 540 nm incubated with Pt-FMO from 0.125 to 1 mg/mL. Inset: the mixtures were centrifuged to visually identify hemoglobin in the supernatant. (D-H) Histological microscopic images. The dissected tumors were stained with H&E (D; scale bar represents 100 µm), Tunel (E; blue fluorescence: Hoechst; green fluorescence: Tunel; scale bar represents 25 µm), Ki67 (F; scale bar represents 25 µm), GPX4 (G; scale bar represents 25 µm) and caspase-3 (H; scale bar represents 25 µm).

The *in vivo* antitumor assay was conducted on tumor-bearing BALB/c-Nude mice. A dosage of 5 mg/kg of various agents, including free cisplatin, free Pt(IV), FMO and Pt-FMO were administrated *via* tail vein injection. It is worth noting that, at this dosage (5 mg/kg), the platinum content in Pt-FMO (0.289 mg/kg) was only 8.89% of that in cisplatin (3.25 mg/kg). Thus, we hypothesized that this low Pt dosage in Pt-FMO can lead to a remarkably reduction in the side effects that arise from platinum agents. The tumor growth curves indicated that Pt-FMO had prominent anti-tumor efficacy, which led to a significant inhibition of tumor growth (Figure 5A). Notably, although the platinum content in Pt-FMO was much lower than that in cisplatin, the antitumor effect of Pt-FMO was still higher. In addition, FMO demonstrated a moderate anti-tumor effect, while administration of Pt(IV) agent showed a negligible effect relative to the PBS group. This result clearly demonstrates the excellent combinatory effect of ferroptotic/apoptotic therapy of Pt-FMO. After treatment, the final tumor can be directly visualized in Figure S18.

In addition to evaluating antitumor efficacy, the potential side effect of various samples was assessed after administration *in vivo*. Based on changes of mouse body weight, the cisplatin group showed significant weight loss, which was due to severe toxicity caused by cisplatin (Figure 5B). In contrast, there was no significant change in body weight in the Pt-FMO treatment group in compared to the PBS group. The minimal weight loss in the Pt-FMO group immediately confirmed our hypothesis that the low Pt dosage of Pt-FMO markedly ameliorates the detrimental effects induced by platinum agents. The biosafety of Pt-FMO can be further validated using the hemolysis assay (Figure 5C). The results suggest that the high blood compatibility of Pt-FMO, which makes the Pt-FMO nanoplatform possible as a potential anti-tumor drug, and can be intravenously administrated. Consistently, the histopathological changes were evaluated using hematoxylin and eosin (H&E) staining. Compared to the control group, treatment with Pt-FMO and cisplatin resulted in significant damage to tumor tissues (Figure 5D). Use of cisplatin also led to numerous cellular alterations on livers, kidneys and lungs, including vacuolar degeneration and disorderly and loose arrangements of the cells, which demonstrates the severe side effects of free cisplatin. However, there was no obvious toxicity on major organs after treatment with Pt-FMO (Figure S19). This result confirms the good biosafety of this drug delivery nanoplatform. Additionally, the drug distribution was evaluated by measuring platinum content in major organs and tumors after 12 h of intravenous drug injection (Figure S20). We discovered that the Pt-drug retention in tumor of Pt-FMO was significantly higher compared to free Pt(IV) and cisplatin, indicating that Pt-FMO is likely to reduce the dosage and frequency of administration, thereby reducing the toxic side effects of drugs.

The *in vivo* therapeutic efficacy of different samples can be also assessed using the terminal deoxynucleotidyl transferase-mediated deoxyuridine triphosphate nick end labeling (Tunel) staining assay and levels of Ki67. The Tunel staining images demonstrated the brightest green fluorescence in Pt-FMO group (Figure 5E), which indicates that the Pt-FMO can effectively induce tumor tissue damage. The significantly reduced Ki67 expression has been noted in Figure 5F, which illustrates the low proliferative property of tumor cells that were treated with Pt-FMO.

In order to further validate the occurrence of both ferroptotic and apoptotic stresses *in vivo*, the expression of GPX4 and caspase-3 were analyzed within tumors. After treatment with Pt-FMO, the number of GPX4 positive cells significantly decreased (Figure 5G), which immediately suggests that the Pt-FMO has substantially induced ferroptotic damage to tumor cells. Moreover, the significant downregulation of GSH can be determined in tumors treated with Pt-FMO (Figure S21). Meanwhile, it has been observed that the expression of caspase-3 has apparently increased post-treatment with Pt-FMO (Figure 5H), which confirms that tumor cell death is associated with the Pt-FMO-mediated apoptosis. These results support that Pt-FMO plays a dual role in both ferroptosis and apoptosis inductors in anti-tumor actions. Therefore, Pt-FMO has been expected to become a promising strategy to initiate ferroptotic and apoptotic combination therapeutics for tumor treatment.

### Magnetic property and MRI performance

The magnetic properties of the FMO were measured in order to assess the potential of FMO particles to be used for magnetic resonance imaging (MRI). Field-dependent magnetization (M-H) curves demonstrated that the saturation magnetization of FO and FMO were 78.45 and 55.00 emu•g^-1^, respectively (Figure S22A), suggesting a strong response of these materials to external magnetic fields. The attraction of Pt-FMO particles to magnets was directly observed (Figure S22B). In addition, nanoparticle magnetization was found to be closely associated with the *T_2_* MRI performance. In general, the higher the magnetization, the stronger the *T_2_* contrast performance is. Hence, a decrease in magnetization caused by deposition of MnCO_3_ is able to weaken the *T_2_* MRI ability of FO. In addition, ferromagnetic particles can easily become magnetized and aggregate unexpectedly under an external magnetic field, which may severely limit their MRI performance and application *in vivo*. Therefore, it is necessary to further analyze the concrete magnetic properties of FO and FMO. It has also become clear that no remanence or coercivity appeared in M-H curves at 300 K. Furthermore, the zero-field cooling (ZFC)/field cooling (FC)-curves of FO and FMO split at low temperatures, while overlaps at high temperatures with no significant blocking temperature in FMO from 3K to 400K (Figure S22C-D). These results demonstrate that the FMO presents typical soft-ferromagnetism, which can avoid the external magnetic field induced aggregation and expect to remain an excellent MRI contrast agent.

**Figure 6:**
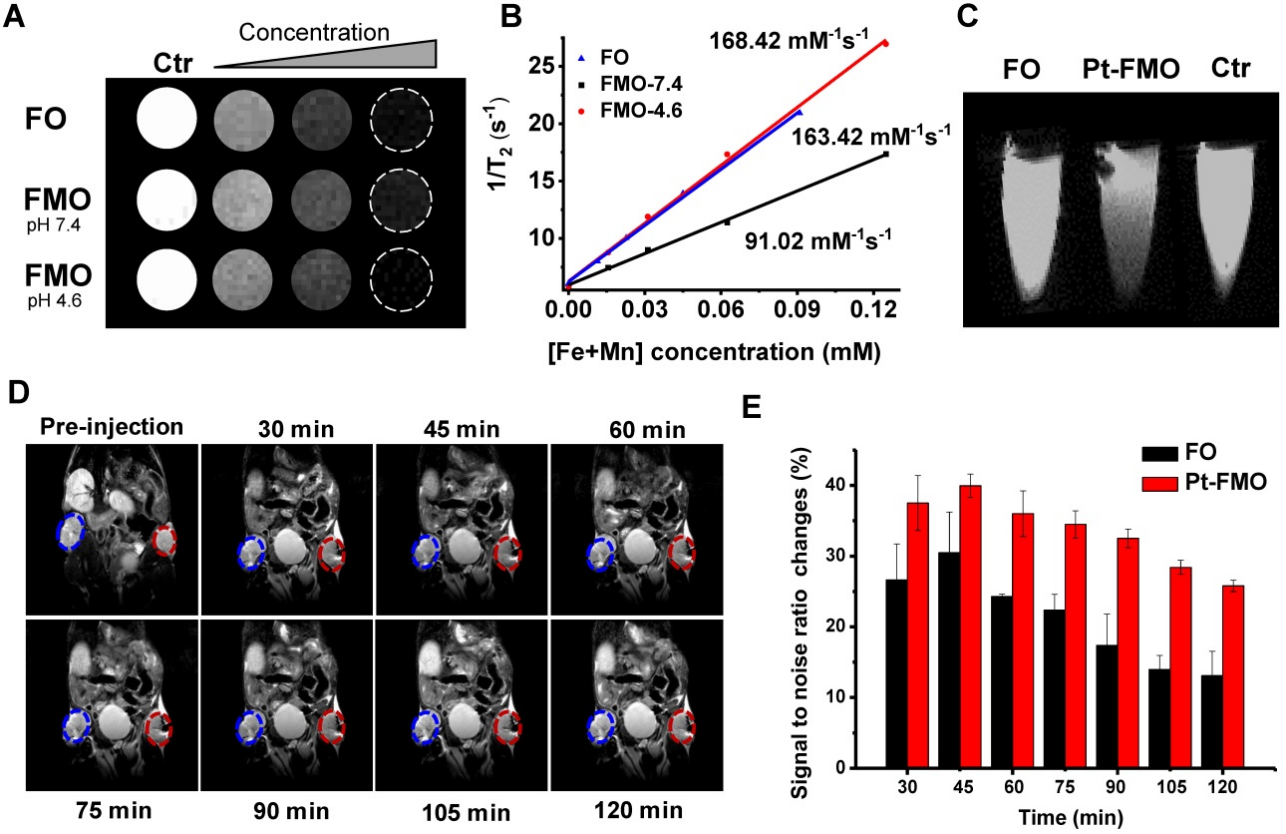
The MRI enhancement nanoparticles *in vitro* and *in vivo*. (A) The *T_2_*-weighted maps of FO and FMO. (B) The transverse relaxation rate (1/*T_2_*) of FO and FMO on the basis of Fe plus Mn concentrations. (C) *T_2_* MR images of HeLa cells post-incubation with FO or Pt-FMOs (5 µg/mL) for 3 h at 37 ^º^C. (D) *T_2_*-weighted MR images of mice after injecting with FO (blue columns) and Pt-FMO (red columns) (2 mg/kg). (E) The corresponding signal-to-noise ratio changes (∆SNR) of Figure (D).

The contrast enhancement property of the nanoparticles was directly evaluated by observing brightness and darkness of MR tubes at different concentrations of FO and FMO. The *T_2_* images of all groups gradually became dark as the concentration of Fe increased (Figure 6A), which suggests the good *T_2_* contrast ability of FMO and FO. Subsequently, we explored their transverse relaxivity by calculating the ratio of 1/*T_2_* to the metal-ion concentration. Results indicated that FMO had significantly lower relaxivity than FO at pH 7.4, suggesting that a deposition of MnCO_3_ is able to weaken the *T_2_* contrast ability of FMO (Figure 6B). Nevertheless, the *T_2_* relaxivity of FMO significantly recovered and reached 168.42 mM^-1^s^-1^ within the acidic condition, which may be attributed to dissolution of MnCO_3_. These results indicate that the MnCO_3_ can be a pH-responsive switch that triggers a greater* T_2_* contrast ability of FMO under the acidic tumor site. Moreover, in order to assess the contrast performance of nanoparticles, the r_2_/r_1_ ratio of FMO has been calculated. The r_2_/r_1_ ratio of FMO was 50.44 under acidic conditions, which is significantly higher than the general r_2_/r_1_ ratio standard (r_2_/r_1_ > 10) of *T_2_*-dominated agents 51, 52. This result immediately suggests that FMO is a *T_2_* MRI contrast agent candidate for tumor diagnosis. Subsequently, a *T_2_*-weighted MRI effect of Pt-FMO was measured *in vitro* and *in vivo* through the use of a 7.0 T MRI scanner to detect the overall performance of Pt-FMO with regards to cellular uptake and bioimaging. In order to make the results more intuitive, only a low concentration of Pt-FMO and FO were added onto cell samples. The cells treated with Pt-FMO exhibited the darkest image compared to cells treated with FO (Figure 6C), suggesting that Pt-FMO has a prominent *T_2_* contrast enhancement for cells, which was in agreement with its MRI performance, as mentioned previously.

Next, Pt-FMO was subcutaneously injected into tumor tissue. It was observed that tumor tissue treated with Pt-FMO demonstrated darker MR images compared to FO, which suggests that the contrast ability of Pt-FMO for tumor detection was greater compared to conventional FO (Figure 6D-E). Correspondingly, the MRI signal-to-noise ratio changes (∆SNR) within tumor regions also validated that the Pt-FMO had higher ∆SNR compared to conventional FO. In addition, approximately 45 min after the injection, the *T_2_*-weighted images of tumor regions treated with Pt-FMO gradually recovered their brightness, which indicated that the body has already begun clearing Pt-FMO. The corresponding ∆SNR values demonstrated similar results. These results reveal that Pt-FMO possesses effective *T_2_* contrast ability *in vivo* and has great potential for clinical use.

## Conclusions

In summary, we designed a Pt-FMO nanoplatform to introduce mutual beneficial cascade reactions to promote ferroptosis and apoptosis in combination with being utilized as an MRI agent. Upon internalization by cancer cells, FMO releases Mn^2+^ and Fe^2+^/Fe^3+^ ions into a weakly acidic microenvironment. Furthermore, the Pt(IV) prodrug is activated through endogenous GSH to generate cisplatin. While Mn^2+^ ions enhance the Fe^2+^/Fe^3+^ ions-induced ferroptosis, cisplatin is capable of elevating cellular H_2_O_2_ levels, which significantly strengthens ferroptosis *via* intracellular Fenton reactions. Meanwhile, the ferroptotic effect of FMO significantly enhances the antitumor effect of cisplatin by providing additional cell-death pathway, in conjunction with apoptosis. Therefore, the Pt-FMO nanoplatform demonstrates a combination effect on tumor cells through cisplatin-induced apoptosis and Fenton-reaction-promoted ferroptosis. A remarkably lower Pt dose (8.89%) of Pt-FMO was able to achieve a significant* in vivo* antitumor effect of cisplatin. Consequently, Pt-FMO demonstrated significantly lower systemic toxicity compared to cisplatin. Moreover, the FMO nanoplatform possesses prominent magnetic properties and high *T_2_* relaxation enhancement, and therefore can be used as an MRI contrast agent for *in vivo* tumor imaging.

## Supplementary Material

Supplementary figures and tables.

## Abbreviations

LPO: lipid hydroperoxides
·OH: hydroxyl radicals
FMO: manganese carbonate-deposited iron oxide
GSH: glutathione
Pt(IV): cisplatin prodrug
Pt-FMO: a cisplatin prodrug-loaded manganese/iron platform
PEI: polyethylenimine
TEM: transmission electron microscopy
DLS: dynamic light scattering
FO: Fe_3_O_4_
TG: thermogravimetric analysis
FT-IR: fourier transform infrared spectroscopy
XPS: X-ray photoelectron spectroscopy
ICP-MS: inductively couple plasma-mass spectrometry
DCF-DA: 2′,7′-dichlorofluorescin diacetate
H&E: hematoxylin and eosin
MRI: magnetic resonance imaging
ZFC: zero-field cooling
FC: field cooling
∆SNR: signal-to-noise ratio changes
