# Supplementary material for: Manganese-deposited iron oxide promotes tumor-responsive ferroptosis that synergizes the apoptosis of cisplatin
Source: Theranostics. 2021 Mar 13;11(11):5418–29. doi: 10.7150/thno.53346 (PMC8039957; doi:10.7150/thno.53346)
Supplement: Supplementary file 1 — Supplementary figures and tables. [file thnov11p5418s1.pdf]

Supporting Information

for

Manganese-deposited Iron Oxide Promotes Tumor-responsive Ferroptosis that Synergizes the Apoptosis of Cisplatin

Materials

$Fe(acac)_3$, $Mn(acac)_2$, polyethyleneimine (PEI), N-hydroxysuccinimide (NHS), N,N′-dicyclohexylcarbodiimide (DCC), N,N-Diisopropylethylamine (DIEA), fluorescein isothiocyanate (FITC), N-acetyl-lcysteine (NAC), Bisbenzimide H 33342 trihydrochloride (Hochest 33342) were purchased from Aladdin Chemical Co. Ltd (Shanghai, China). Cisplatin was purchased from Shandong Boyuan Pharmaceutical Co., Ltd (Shandong, China). An average molecular weight of 5000 Da $mPEG_{5k}$-NH_2 and $mPEG_{5k}$-COOH (PEG = polyethylene glycol) were purchased from Shanghai Pengsheng Biotech Development Co. Ltd. (Shanghai, China). 5,5-dimethyl-1-pyrroline n-oxide (DMPO) were bought from Dojindo (Dojindo, China). Deferoxamine mesylate salt (DFO) was purchased from Sigma-Aldrich. Sorafenib (SRF) was purchased from Energy Chemical Co. Ltd (Shanghai, China). Dulbecco's modified eagle medium (DMEM), Penicillin-Streptomycin Solution were purchased from Gibcol Solutions Ltd (Auckland, New Zealand). Fetal bovine serum was purchased from lonsera (USA). Trypsin, 5-diphenyltetrazolium bromide (MTT), BCA protein assay kit, reactive oxygen species assay kit, GSH and GSSG assay kit, apoptotic DNA ladder isolation kit, cell lysis buffer for Western Blotting and IP, 4′, 6-diamidino-2-phenylindole (DAPI), primary antibody dilution buffer and other commonly used buffers were purchased from Beyotime (Jiangsu, China). GSH detection kit and H_2O_2 detection kit was purchased from Jiancheng (Jiangsu, China). HRP-conjugated affinipure goat anti-mouse IgG(H+L) was purchased from Proteintech Group Co. (Illinois, USA). Anti-GPX4 antibody, HRP-labeled Goat Anti-Rabbit IgG(H+L) were purchased from Abcam plc. (Cambridge, UK). Anti-AIF, Bax, Bcl-2 and actin antibody were purchased from Santa Cruz Biotechnology (CA, USA). Polyvinylidene fluoride was purchased from Millipore (Maryland, USA). BODIPY581/591-C11, SuperSignal West Pico PLUS chemiluminescent substrate was purchased from Thermo Fisher Scientific Co. (Maryland, USA). Other chemicals and reagents were purchased from Sinopharm Chemical Reagent Co. Ltd. All chemicals and reagents

were used without further purification. The water used in all experiments was ultra-pure MilliQ water (18 MΩ).

Experimental details

Characterization. The morphology and element mapping of particles were observed by transmission electron microscope (JEM-ARM200F, JEOL Co., Japan and Talos F200X, JEOL Co., USA). The size distribution of particles was measured via DLS detector (NanoBrook 90Plus PALS, Brookhaven Co., USA). The crystal structure measurement was performed on XRD (TTR-III, Rigaku Co., Japan). The measurements of composition and structure of samples were conducted on XPS (ESCALAB 250, Thermo-VG Scientific Co., USA) and FT-IR spectrometer (TENSOR 27, Brookhaven Co., USA). Magnetic Property was measured by magnetic property measurement system (MPMS3, Quantum Design Inc., USA).

Synthesis of manganese-deposited iron oxide nanoparticles. The equal mixture of Fe(acac)$_3$ and Mn(acac)$_2$ (1 g) were added into ethylene glycol (20 mL) and diethylene glycol (80 mL), under continuous stirring at 80 ^{o}C for 40 min. Afterward, PEI (3 g) was added. After stirring for 30 min, 10 mL of triethanolamine was added into the above solution with vigorous stirring for an additional 30 min. The obtained solution was transferred to an autoclave and maintained at 200 ^{o}C overnight. Finally, the black solid products were collected using external magnet, and washed more than three times with distilled water and alcohol, respectively.

Synthesis of Pt(IV) (c,c,t-[Pt(NH$_3$)$_2$Cl$_2$(OOCCH$_2$CH$_2$COOH)$_2$]). Oxoplatin was prepared following a literature procedure, described briefly here. H_2O_2 (37.5 mL, 30% w/v) was added to the suspension of 900 mg cisplatin (3 mmol) in 7.5 mL water. The mixture was stirred for 1.5 h at 50 ^{o}C and subsequently allowed to cool to room temperature for 12 h. The solution was lyophilized and precipitate was washed with cold water, acetone and ether, and dried again.

For the synthesis of c,c,t-[Pt(NH$_3$)$_2$Cl$_2$(OOCCH$_2$CH$_2$COOH)$_2$], 0.9 g Succinic anhydride (4.5 mmol)

was added to 10 mL N,N-Dimethylformamide (DMF) solution of oxoplatin (300 mg, 0.9 mmol). The mixture was stirred overnight at room temperature. Then the resultant was washed with chloroform and ether to yield a pale yellow solid. The product was dried and was characterized by ^{1}H NMR.

Preparation of Pt(IV) drug-loaded Pt-FMO. First, manganese-deposited iron oxide nanoparticles (100 mg) were submerged into an alcohol (300 mL) solution containing PEI (1 g), and stirred for 12h. The mixture was then shaken for 30 min at 45°C. The particles were collected using an external magnet and washed with anhydrous ethanol and DMSO, respectively. In addition, 1 g $mPEG_{5K}$-COOH, 80 mg DCC, 44.8 mg NHS and 64 µL DIEA were dissolved into a 40 mL DMSO solution of nanoparticles, and stirred for 12 h. After, the particles were collected and washed, and then dispersed into anhydrous ethanol for future use.

Preparation of PEGylated manganese-deposited iron oxide nanoparticles. Firstly, manganese-deposited iron oxide nanoparticles (100 mg) was dispersed into alcohol (300 mL) solution containing PEI (1 g) and stirred for 12h. The mixture was shaken 30 min at 45 ^{o}C. The particles were collected by external magnet and washed with anhydrous ethanol and DMSO, respectively. In addition, 1 g MPEG5K-COOH, 80 mg DCC, 44.8 mg NHS and 64 µL DIEA were dissolved into 40 mL DMSO solution of nanoparticles and stirred for 12 h. After that, the obtained particles were collected and washed, then dispersed into anhydrous ethanol for further use.

Preparation of FMO-FITC. Firstly, manganese-deposited iron oxide nanoparticles (25 mg) was dispersed into alcohol (75 mL) solution containing PEI (0.33 g) and stirred for 12 h. The mixture was shaken 30 min at 45 ^{o}C. The particles were collected by external magnet and washed with ethanol and H_2O, respectively. In addition, 15 µmol FITC and 0.15 µmol trimethylamine were dissolved into 10 mL H_2O solution of nanoparticles and stirred for 12 h. After that, the obtained particles were collected and washed, and then 0.25 g MPEG5K-NH2, 20 mg DCC, 11.2 mg NHS and 17 µL DIEA were dissolved into 10 mL of DMSO and stirred for another 12 h. Finally, the product was collected and washed at least three times and dispersed into water for further use.

Accumulated release profiles of Fe, Mn and Pt elements. 50 mg Pt-FMO were uniformly dispersed into PBS (pH 7.4) and shaken at 37 °C. The supernatant was collected at desirable time points, and the Fe, Mn and Pt concentrations were simultaneously determined by ICP-OES (ICAP-7200, Thermo Fisher Scientific Co., USA). The same release experiments were done at pH 4.6 using acetate buffered solution.

ROS detection assay using ESR. In a typical experiment, 100 μg mL^{-1} Pt-FMO was added into the buffer solution (pH 4.6, 6.5 and 7.4, respectively) containing 1.0 mM H_2O_2 and 100 μM DMPO. After violent ultrasonication for 60 s, the mixture was transferred to a quartz tube for ESR assay.

Observations of cellular uptake behavior. HeLa or HepG2 cells were seeded in six-well culture plates at a density of 5×10^4 cells/well. After cultured in 5% CO_2 at 37 °C overnight, FMO-FITC was added and Incubated for different time points. Subsequently, the cells were washed with PBS three times and stained by DAPI or Hoechst at 37 °C in dark condition for 10 min. The samples were analyzed using fluorescence microscope (U-HGLGPS, Olympus Co., Japan) for FITC.

Measurement of Pt-DNA adduct formation. For the measurement of Pt concentration in cellular DNA in HeLa cells, the cells were seeded in six-well culture plates at a density of 5×10^4 cells/well. After cultured in 5% CO_2 at 37 °C overnight, Pt-FMO, cisplatin and Pt(IV) were added and incubated for 4 h. DNA was isolated by using Genomic DNA Mini Preparation Kit, and Pt content in DNA was analyzed using ICP-MS (PlasmaQuad 3, Thermo Fisher Scientific Co., USA).

Measurement of cellular ROS generation. HeLa cells were seeded in six-well culture plates at a density of 5×10^4 cells/well. After cultured in 5% CO_2 at 37 °C overnight, different drugs were added and Incubated for 6h. Subsequently, the cells were washed with PBS three times, used the reactive oxygen species assay kit and followed the product instructions to determine ROS levels. The samples were analyzed using fluorescence microscope for FITC.

***In vitro* cellular cytotoxicity assays.** HeLa or HepG2 cells were seeded in 96-well plates at 5000 cells/well in 100 μL of complete medium, and Incubated in a 5% CO_2 atmosphere at 37 °C for 24 h. The culture medium was then replaced with 100 μL of freshly prepared culture medium containing drugs at different concentrations. The cells were further Incubated for 48 h, and then the medium in each well was replaced with fresh culture medium and the MTT solution was added. The cells

were incubated for another 4 h to allow viable cells to reduce the yellow tetrazolium salt (MTT) into dark blue formazan crystals. Finally, 150 µl of DMSO was added to the wells and Incubated for 20 min at 37°C. The absorbance was measured at 490 nm by using a microplate reader (680 Microplate Reader, Bio-Rad, USA). For live/dead cell staining assay, cells were seeded in 6-well plates at 2×10^5 cells per well in 2 mL of DMEM containing 10% FBS and incubated for 24 h before treatment. After that, the culture medium was replaced with freshly prepared culture medium containing drugs. After another 72 h, the cells were washed for three times with PBS, followed by staining with FDA (10 μM) and PI (20 μM). After 20 minutes of incubation, the medium was removed and the cells were washed three times with PBS. The fluorescence imaging of cells was analyzed by fluorescence microscopy.

Intracellular lipid peroxide measurement. For the measurement of intracellular lipid peroxide in HeLa cells, the cells were seeded in six-well culture plates at a density of 5×10^4 cells/well. After cultured in 5% CO_2 at 37 °C overnight, the culture medium was then replaced with freshly prepared culture medium containing different drugs for further 8 h. Subsequently, the cells were washed with PBS three times and stained by BODIPY581/591 at 37°C in dark condition for 30 min. The intracellular lipid peroxide content was determined by fluorescence microscope.

Comparison of the cellular GSH depletion rate. HeLa cells were seeded in six-well culture plates at a density of 2×10^5 cells/well. After cultured in 5% CO_2 at 37°C overnight, the culture medium was then replaced with freshly prepared culture medium containing different drugs. The cells were further incubated for 48 h, and harvested to determine cell number. Nearly 6×10^4 live cells from each sample were transferred to new tubes, washed with PBS and centrifuged at 1,200 rpm at 4 °C for 5 min three times. Then used the GSH and GSSG Assay Kit and followed the product instructions to determine GSH levels. For *in vivo* assay, 5 mg/kg Pt(IV), FMO and Pt-FMO have been intratumoral injected into the mice. The GSH level was detected using GSH detection kit after 24 h.

The H_2O_2 level *in vitro* and *in vivo*. HeLa cells were seeded in six-well culture plates. After cultured in 5% CO_2 at 37 °C overnight, the culture medium was then replaced with freshly prepared culture medium containing different concentration of cisplatin. After further incubating for 24 h, the cells were harvested. The H_2O_2 level was detected using H_2O_2 detection kit. For *in vivo* assay, 5 mg/kg

cisplatin has been intratumoral injected into the mice. The H_2O_2 level was detected using H_2O_2 detection kit after 24 h.

Microscopy images of Pt-FMO treated cells. HeLa cells were seeded in 60mm culture dish plates at a density of 2×10^6 cells/well. After cultured in 5% CO_2 at 37 ^oC overnight, the culture medium was then replaced with freshly prepared culture medium containing different drugs and further Incubated for 24 h. The cell samples were fixed with 2.5% glutaraldehyde stationary liquid and stained with lead citrate and uranyl acetate, collected on copper grids, and finally analyzed using transmission electron microscope.

Western blot analysis. HeLa cells were seeded in 35 mm culture dish plates at a density of 1×10^6 cells/well. After cultured in 5% CO_2 at 37 ^oC overnight, the culture medium was then replaced with freshly prepared culture medium containing different drugs, and further Incubated for 24 h. After washing the cells with cold PBS three times, using the Cell lysis buffer for Western and IP and followed the product instructions to extract the total proteins from cultured cells. Protein concentrations were determined by using the BCA protein assay kit. About 70 μg protein samples were denatured in 5 × sodium dodecyl sulfate polyacrylamide gel electrophoresis (SDS-PAGE) sample buffer, and subjected to SDS-PAGE on 15% tris-glycine gels. The separated proteins were transferred onto 0.45 μm PVDF membranes for 1 h at 200 mA. The membranes were blocked with 5% nonfat milk (w/v) in TBST for 1 h at room temperature. The specific primary antibodies (dilution by primary antibody dilution buffer) were used to probe the protein levels of the different desired molecules overnight at 4 °C, followed by appropriate peroxidase-conjugated secondary antibodies (dilution by 5% nonfat milk) for 2 h at room temperature. The blots were visualized using Super Signal West Pico PLUS chemiluminescent substrate and analyzed using chemiluminescence imaging system (Image Quant LAS 4000, GE Healthcare, USA).

DNA laddering assay. DNA laddering assay was used to evaluate the DNA damage in HeLa cells after treatment with different drugs for 24 h. The DNA was extracted according to the user manual provided by the manufacturer of the apoptotic DNA ladder isolation kit, and images were captured after gel electrophoresis with 1% agarose gel.

***In vivo* antitumor assay.** The HeLa single-cell suspension in PBS (5×10^6 per mouse) was injected subcutaneously into the buttock of the mouse. When the tumor grew to a size of 80-150 mm^3, the 25 mice were randomly divided into five treatment groups (PBS, cisplatin, Pt(IV), FMO and Pt-FMO), with all drugs administered intravenously via tail vein. The drugs were given 8 times at 2-day intervals. Tumor growth was monitored by measuring the perpendicular diameter of the tumor using calipers every two days and calculated according to the formula: tumor volume (mm^3) = $0.5 \times \text{length} \times \text{width}^2$. Five days after the last treatment, animals were sacrificed and tumors were excised for immunohistochemistry analysis. For the biodistribution assay, 10 mg/kg cisplatin, Pt(IV) and Pt-FMO has been tail vein injected into mice, respectively. After 12 h, animals were sacrificed, then tumors and major organs were excised for the ICP-MS analysis.

MR experiment *in vitro* and *in vivo.* All MR studies were performed on a 7.0 T scanner (Bruker Co., Ltd, Germany) using a volume RF coil. For cell phantom or tube samples, a series of spin echo images were acquired for transverse relaxation time (T_2) measurement, identical in all aspects (repetition time (TR) 2500 ms, effective echo time (TE) 5.6 ms, band width (BW) 25 kHz, slice thickness 1 mm, matrix 96 × 96, 3 average) except for 20 echoes time (TI) which was varied linearly from 10 to 2500 ms. For the *in vivo* MR studies, T_2-weighted MR images of tumor were acquired with the axial and coronal orientation using a fast spin-echo sequence. The following acquisition parameters were chosen: repetition time (TR) = 370 ms, echo time (TE) = 11.6 ms, field of view (FOV) = 40 mm × 40 mm, matrix size = 192 × 192, slice thickness = 1 mm (12 slices, gap = 0), 1 average, and bandwidth (BW) = 50 kHz.

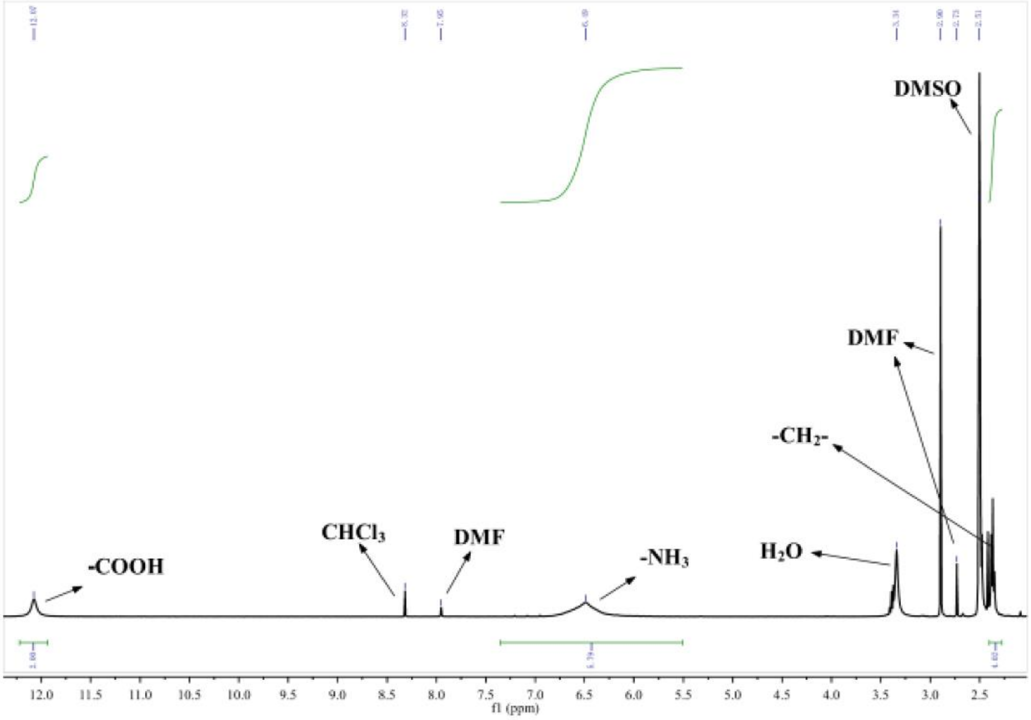

Figure S1. ^{1}H NMR spectrum of Pt(IV) (*c,c,t*-[$Pt(NH_3)_2Cl_2(OOCCH_2CH_2COOH)_2$]) in $(CD_3)_2SO$. One of the -CH_2- signals overlaps with DMSO.

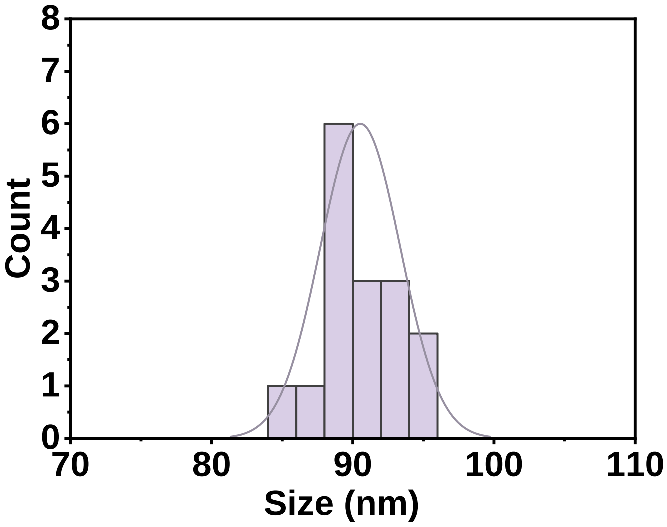

Figure S2. The particle size distribution histogram of Figure 1A.

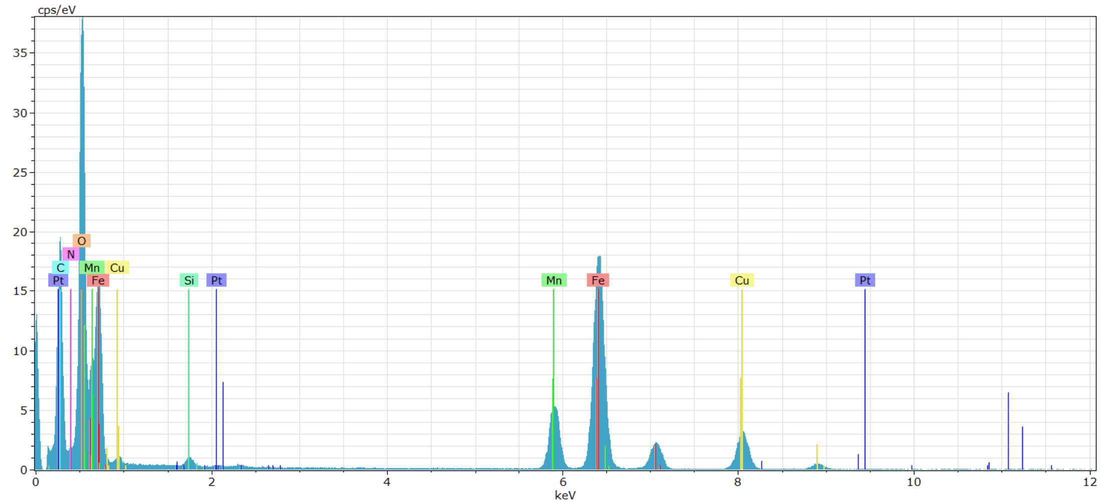

Figure S3. The energy dispersive X-ray spectrum of Pt-FMO.

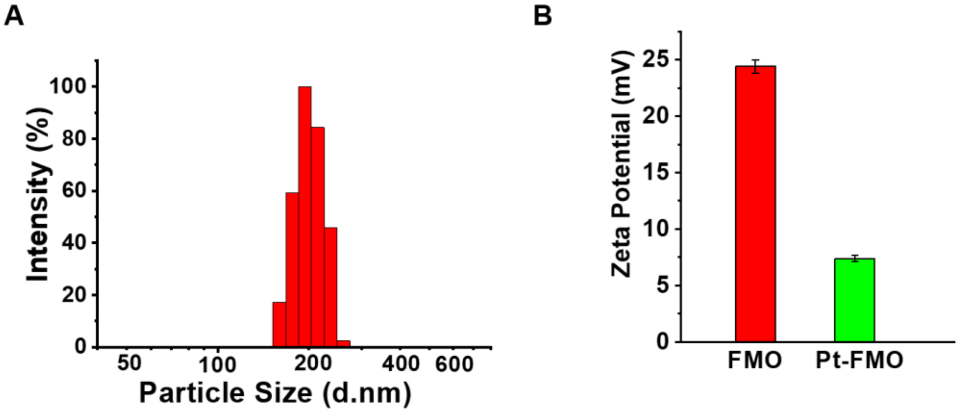

Figure S4. Hydrodynamic size and zeta potential of nanoparticles. (A) Hydrodynamic size distributions of Pt-FMO. (B) Zeta potential of FMO and Pt-FMO.

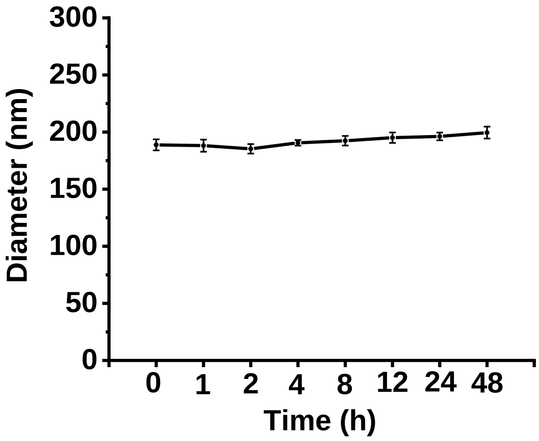

Figure S5. Stability of Pt-FMO in DMEM with 10% FBS. Particle sizes were measured by DLS with different incubation times.

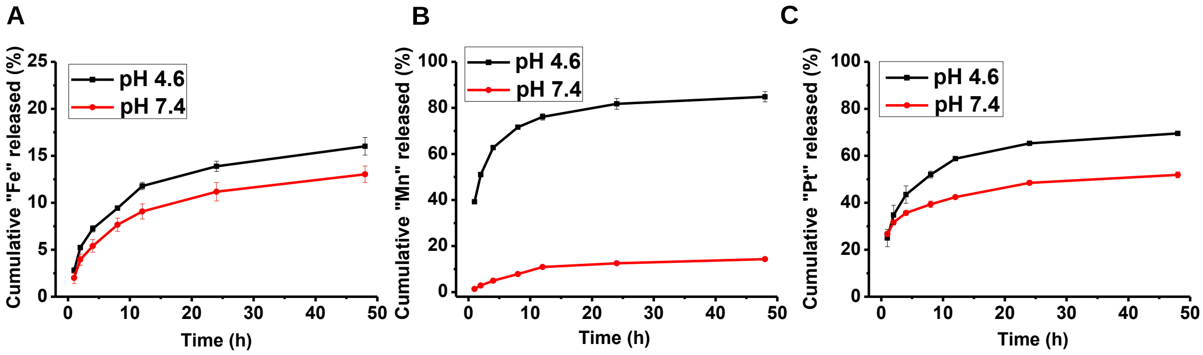

Figure S6. The release behavior of Pt-FMO. (A) Fe; (B) Mn; (C) Pt. Measurements were performed in neutral (pH 7.4, red lines) or acidic pH (pH 4.6, black lines).

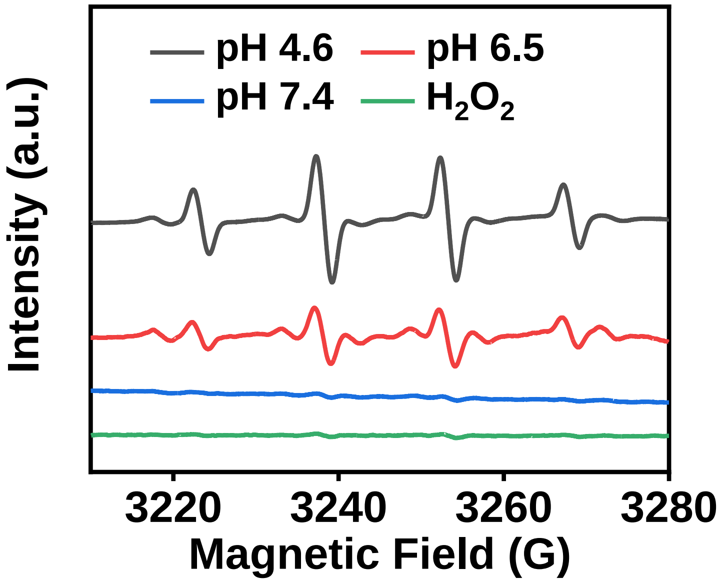

Figure S7. ESR spectrum of •OH trapped by DMPO (Pt-FMO: 100 μg/mL, H_2O_2: 1.0 mM).

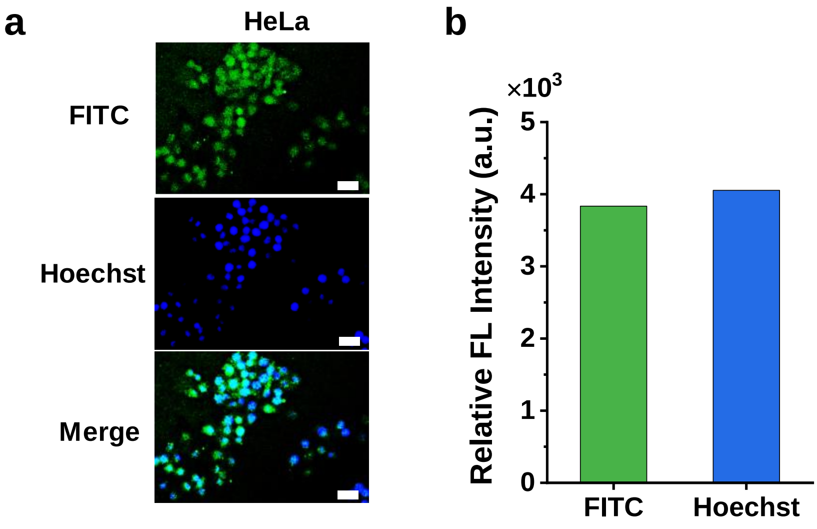

Figure S8. Cellular uptaking of FMO-FITC by HeLa cells. (a) Fluorescence microscopy images (FITC: green fluorescence for FMO-FITC; Hoechst: blue fluorescence for cell nucleus). (b) Quantification of the relative FL intensity of FITC and Hoechst. The cells were incubated with FMO-FITC for 4 h in 37 °C.

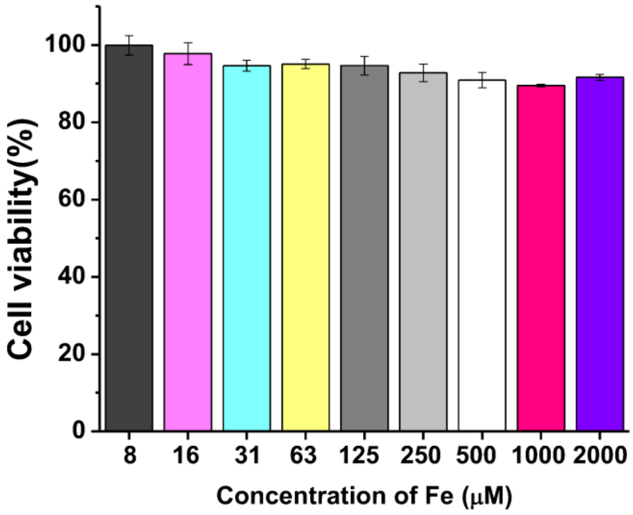

Figure S9. Cell viability of HeLa cells after the treatment with FO for 48 h in 37 °C.

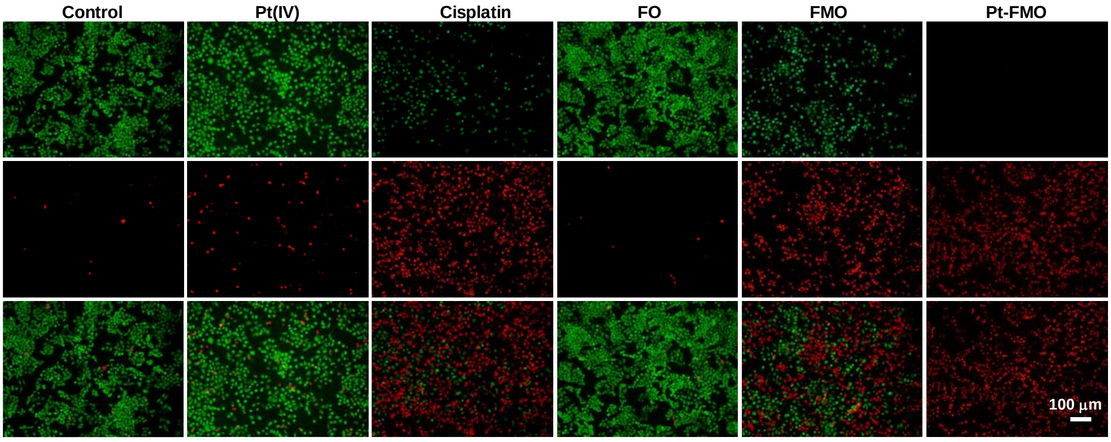

Figure S10. Fluorescence imaging of live/dead cells. HeLa cells were treated with Pt(IV), cisplatin, FO, FMO and Pt-FMO at an equivalent concentration of 5 μM Pt for 72h and stained with 5 μM FDA (green for live cells) and 10 μM PI (red for dead cells). The concentration of Mn in FMO was consist with that in Pt-FMO (scale bar = 100 μm).

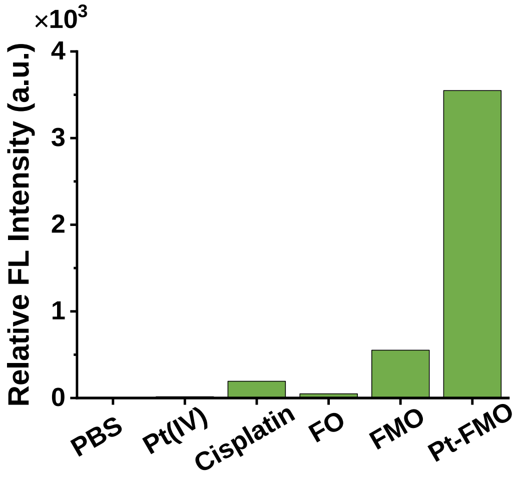

Figure S11. Quantification of the ROS level in HeLa cells with DCFH-DA staining. Cells were incubated with different formula at an equivalent Pt dosage of 15 μM for 4 h at 37 °C. The concentration of Fe and Mn in FO and Mn was consistent with Pt-FMO.

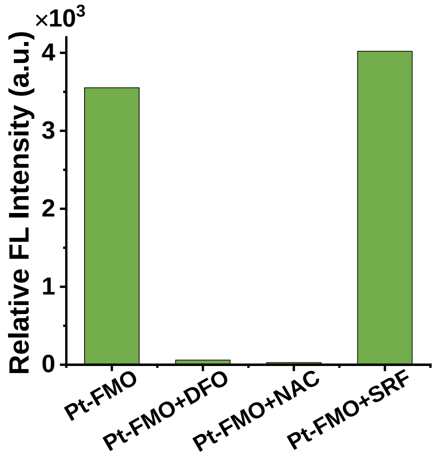

Figure S12. Quantification of the ROS level in HeLa cells with DCFH-DA staining. Cells were incubated with different formula at an equivalent Pt dosage of 15 μM for 4 h at 37 °C. DFO (100 μM) and NAC (3 mM) were used separately to pretreat cells for 1 h. SRF (3 μM) and Pt-FMO was added in the same time.

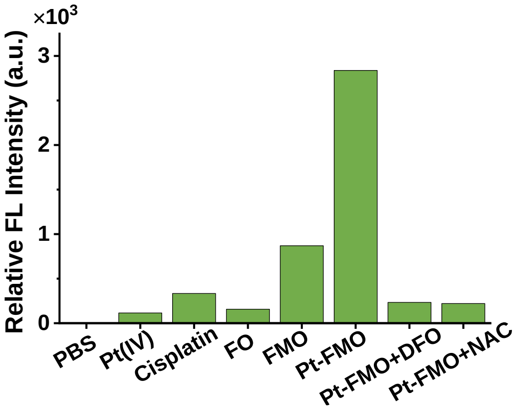

Figure S13. Quantification of the LPO level in HeLa cells with BODIPY-C11 staining. Cells were incubated with different formula at an equivalent Pt dosage of 15 μM for 4h at 37 °C. The concentration of Fe and Mn in FO and Mn was consistent with Pt-FMO. DFO (100 μM) and NAC (3 mM) were used separately to pretreat cells for 1 h.

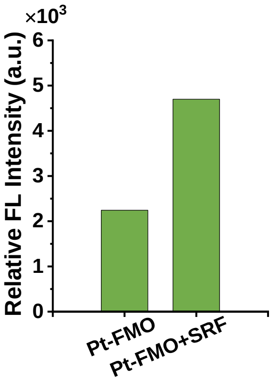

Figure S14. Quantification of the LPO level in HeLa cells with BODIPY-C11 staining. Cells were incubated with different formula at an equivalent Pt dosage of 15 μM for 4 h at 37 °C. SRF (3 μM) and Pt-FMO was added in the same time.

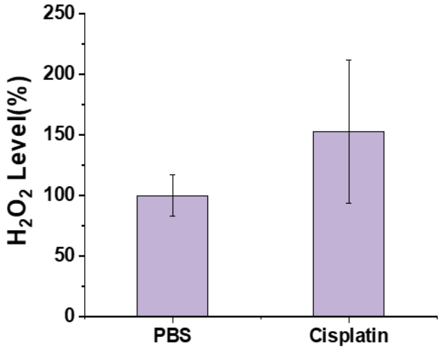

Figure S15. The H_2O_2 level *in vivo* after treating with cisplatin for 24 h. The concentration of cisplatin: 5 mg/kg.

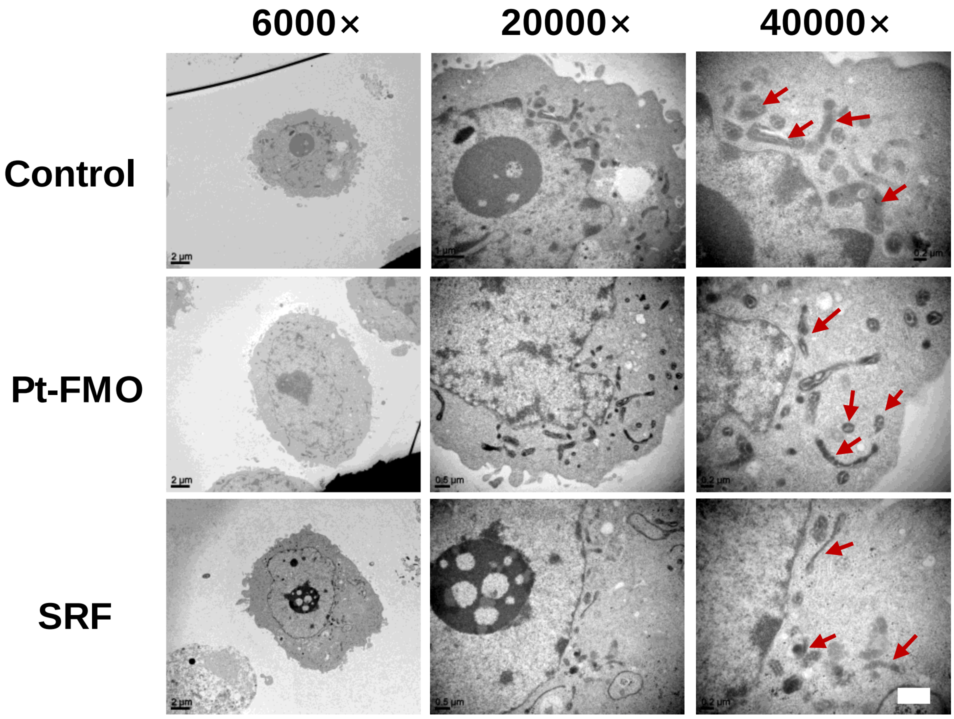

Figure S16. Morphology of cells treated with PBS, free Pt(IV), Pt-FMO or SRF for 24 h at 37 °C. Free Pt(IV) and Pt-FMO were at an equivalent Pt dosage of 10 μM. SRF dosages used in this study was 3 μM. The enlarged regions show the shape change of mitochondria. Scale bar: 0.5 μm.

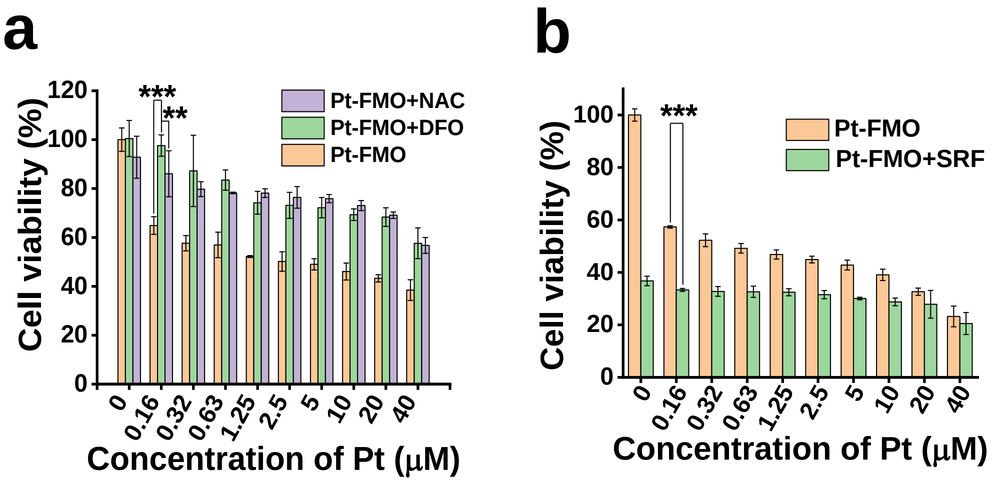

Figure S17. Cell viability of HeLa cells after the treatment with (a) Pt-FMO, Pt-FMO+DFO, Pt-FMO+NAC and (b) Pt-FMO+SRF for 48 h at 37 °C, respectively. DFO (100 μM) and NAC (3 mM) m were used separately to pretreat cells for 1 h before cells treated with Pt-FMO. The concentrations of SRF in all Pt-FMO+SRF samples were 3 μM. n = 6; *p < 0.05, **p < 0.01, ***p < 0.001. Statistical analysis was performed using the t-test.

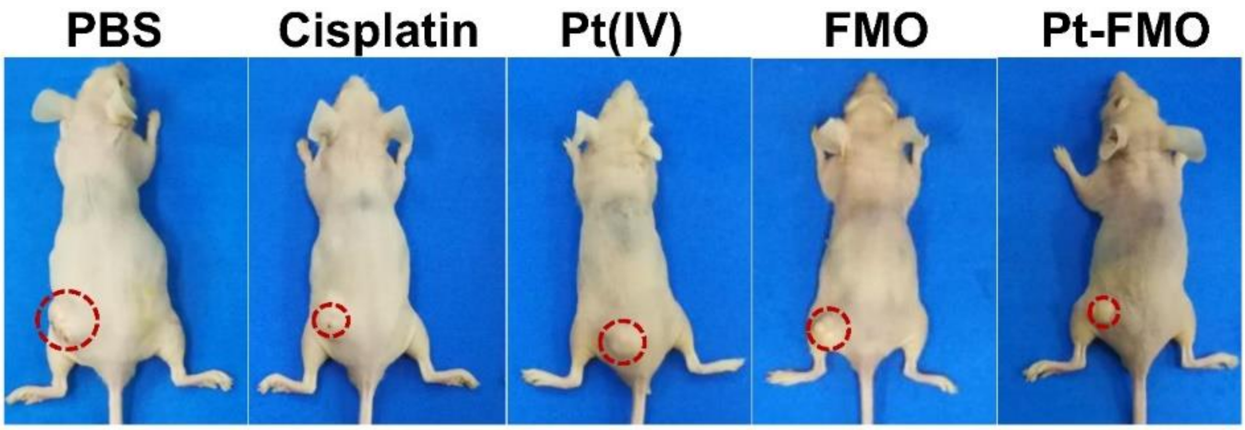

Figure S18. Nude mice photographs at the end of treatment. The tumor locations were indicated by circles.

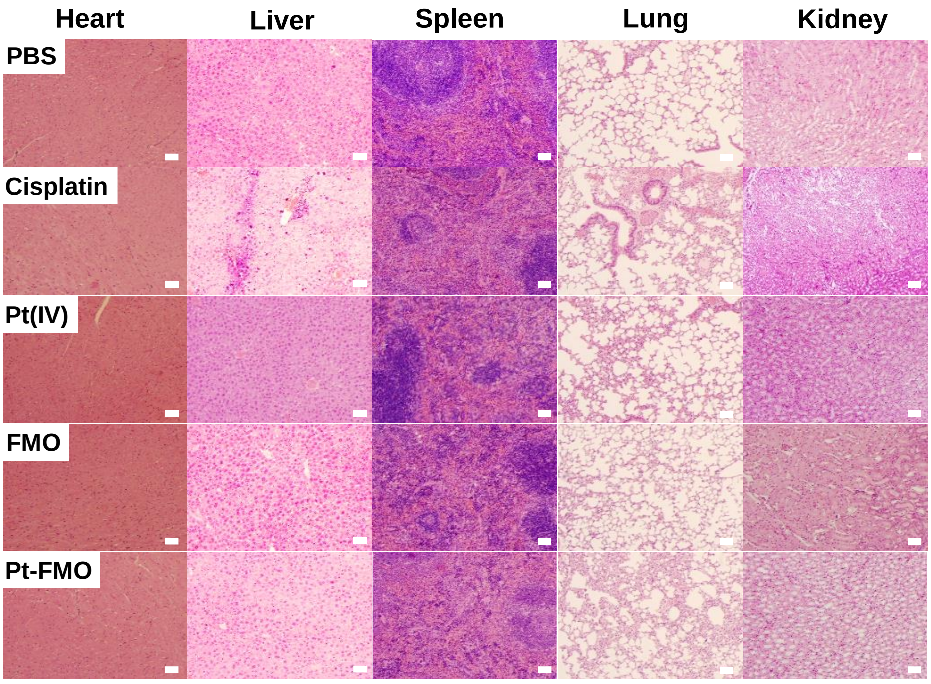

Figure S19. Histological microscopic images stained with H&E (Scale bar: 100 μm).

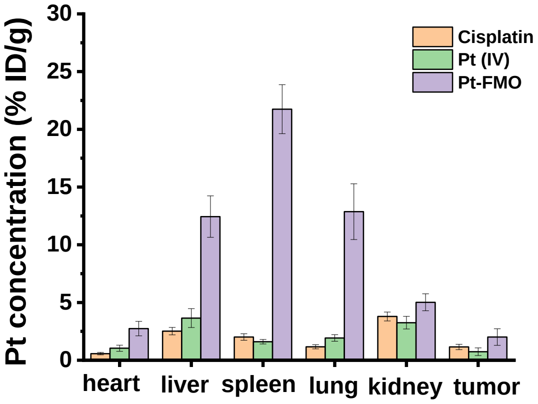

Figure S20. The biodistribution of Pt in tumors and major organs treated with cisplatin, Pt(IV) and Pt-FMO for 12 h. The dosage was 10 mg/kg via tail vein injection.

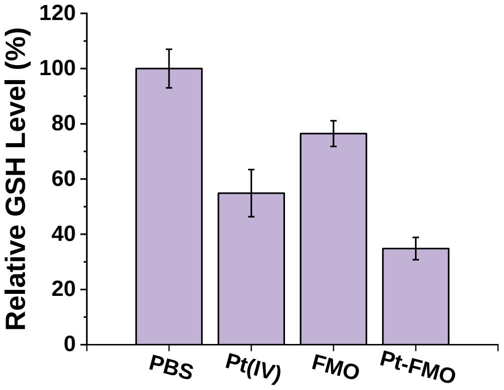

Figure S21. The relative GSH level *in vivo* treated with Pt(IV), FMO and Pt-FMO for 24 h.

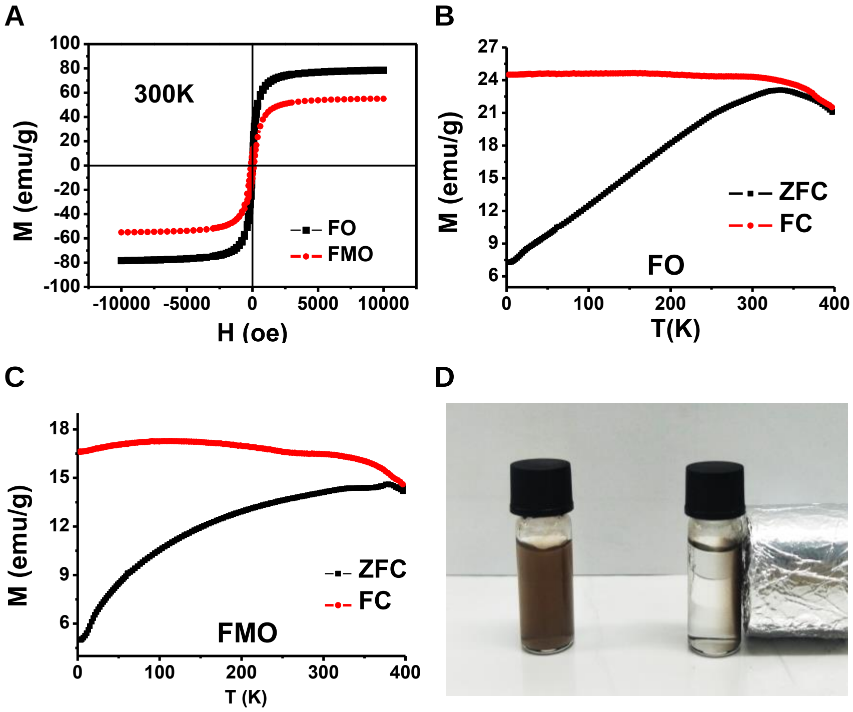

Figure S22. The measurement of magnetic property of nanoparticles. (A) Hysteresis loops of FO and FMO at 300 K. (B) Photography of Pt-FMO in aqueous solution in the absence (left) or presence (right) of magnet. (C-D) The temperature dependence of ZFC and FC magnetization of FO (C) and FMO (D).
